# Genetic Microbial Source Tracking Support QMRA Modeling for a Riverine Wetland Drinking Water Resource

**DOI:** 10.3389/fmicb.2021.668778

**Published:** 2021-07-14

**Authors:** Julia Derx, Katalin Demeter, Rita Linke, Sílvia Cervero-Aragó, Gerhard Lindner, Gabrielle Stalder, Jack Schijven, Regina Sommer, Julia Walochnik, Alexander K. T. Kirschner, Jürgen Komma, Alfred P. Blaschke, Andreas H. Farnleitner

**Affiliations:** ^1^Institute of Hydraulic Engineering and Water Resources Management, TU Wien, Vienna, Austria; ^2^Research Group Environmental Microbiology and Molecular Diagnostics E166/5/3, Institute of Chemical, Environmental and Bioscience Engineering, TU Wien, Vienna, Austria; ^3^Institute for Hygiene and Applied Immunology, Medical University of Vienna, Vienna, Austria; ^4^Institute of Wildlife Ecology, University of Veterinary Medicine, Vienna, Austria; ^5^Department of Statistics, Informatics and Modelling, National Institute for Public Health and the Environment (RIVM), Bilthoven, Netherlands; ^6^Faculty of Geosciences, Department of Earth Sciences, Utrecht University, Utrecht, Netherlands; ^7^Institute of Specific Prophylaxis and Tropical Medicine, Medical University of Vienna, Vienna, Austria; ^8^Division Water Quality and Health, Department of Pharmacology, Physiology, and Microbiology, Karl Landsteiner University of Health Sciences, Krems an der Donau, Austria

**Keywords:** genetic microbial source tracking markers, microbial fate and transport model, hydrodynamic model, *Cryptosporidium*, *Giardia*, QMRA, microbial decay in environment

## Abstract

Riverine wetlands are important natural habitats and contain valuable drinking water resources. The transport of human- and animal-associated fecal pathogens into the surface water bodies poses potential risks to water safety. The aim of this study was to develop a new integrative modeling approach supported by microbial source tracking (MST) markers for quantifying the transport pathways of two important reference pathogens, *Cryptosporidium* and *Giardia*, from external (allochthonous) and internal (autochthonous) fecal sources in riverine wetlands considering safe drinking water production. The probabilistic-deterministic model QMRAcatch (v 1.1 python backwater) was modified and extended to account for short-time variations in flow and microbial transport at hourly time steps. As input to the model, we determined the discharge rates, volumes and inundated areas of the backwater channel based on 2-D hydrodynamic flow simulations. To test if we considered all relevant fecal pollution sources and transport pathways, we validated QMRAcatch using measured concentrations of human, ruminant, pig and bird associated MST markers as well as *E. coli* in a Danube wetland area from 2010 to 2015. For the model validation, we obtained MST marker decay rates in water from the literature, adjusted them within confidence limits, and simulated the MST marker concentrations in the backwater channel, resulting in mean absolute errors of < 0.7 log_10_ particles/L (Kruskal–Wallis *p* > 0.05). In the scenarios, we investigated (i) the impact of river discharges into the backwater channel (allochthonous sources), (ii) the resuspension of pathogens from animal fecal deposits in inundated areas, and (iii) the pathogen release from animal fecal deposits after rainfall (autochthonous sources). Autochthonous and allochthonous human and animal sources resulted in mean loads and concentrations of *Cryptosporidium* and *Giardia* (oo)cysts in the backwater channel of 3–13 × 10^9^ particles/hour and 0.4–1.2 particles/L during floods and rainfall events, and in required pathogen treatment reductions to achieve safe drinking water of 5.0–6.2 log_10_. The integrative modeling approach supports the sustainable and proactive drinking water safety management of alluvial backwater areas.

## Graphical Abstract

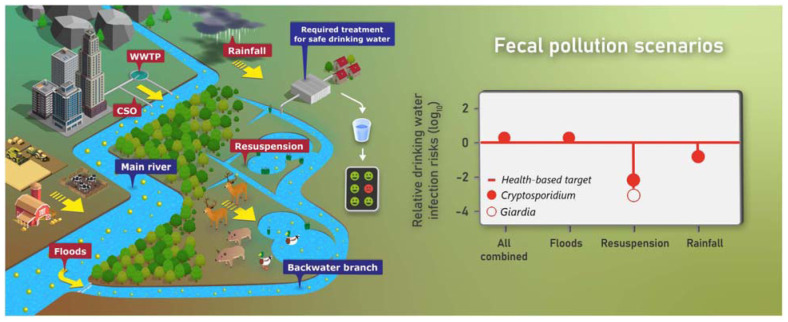

## Introduction

Alluvial backwater areas along large rivers are important as natural habitats, for flood protection, and contain valuable resources for drinking water production. The impacts of urban wastewater discharges, and livestock and wildlife fecal deposits pose substantial hazards to these ecosystems concerning water supply (Hogan et al., 2012; Frick et al., 2020). River water impacted by human wastewater and/or diffusive animal sources may transport fecal pathogens into the backwater area from outside (allochthonous sources). Inside the backwater area, pathogens may be released from animal fecal deposits following rainfall events, or may be resuspended in inundated areas during floods (autochthonous sources). Several authors have studied the impact of allochthonous and anthroponotic pathogens, such as human-specific viruses, on the microbiological quality of wetland water resources considering safe drinking water production (Sanders et al., 2005; Daniels et al., 2014; Liu et al., 2015; Derx et al., 2016). In this respect, however, there is little known about the relative contribution of animal versus human and autochthonous versus allochthonous sources of fecal pollution.

The microbial transport and removal mechanisms in wetlands have been primarily studied for fecal indicator organisms (FIO) (Sanders et al., 2005; Liu et al., 2015). Solely relying on FIO, however, does not allow differentiating the impact of different sources, as FIO occur in all human and animal sources. Microbial source tracking markers (MST) provide therefore immensely valuable information to identify fecal pollution sources for wetlands. This was demonstrated, e.g., by Frick et al. (2020), who linked FIO data with host-associated MST data and river connectivity in an alluvial Danube wetland. If combined with pathogen data, host-associated microbial source tracking markers of sufficient specificity and sensitivity can greatly support the source-targeted calibration of microbial fate and transport models and support a health risk assessment, as shown by Derx et al. (2016) and Demeter et al. (2021). So far, the spectrum of available animal and human MST marker assays has not yet been exploited in this context. In combination with FIO and zoonotic pathogen data, host-associated MST markers could significantly support microbial fate and transport modeling and microbial infection risk assessments in wetlands.

Important transport processes in wetlands are the advection, release and resuspension, decay and settling of microbial particles or chemical substances (Pavlik et al., 1999; Grant et al., 2001; Hogan et al., 2012; Peterson and Hanna, 2016). Further influencing factors for the retention mechanisms are water temperature, turbidity, salinity, and vegetation cover (Daniels et al., 2014). The flow patterns driving these transport processes in riverine or coastal wetlands vary in space and time, and are commonly simulated based on multi-dimensional, hydrodynamic flow and transport models (Sanders et al., 2005;

Liu et al., 2015). Due to the uncertainty of the source and transport variables, several studies conducted in wetlands used probabilistic-deterministic approaches to model microbial fate and transport. Daniels et al. (2014) developed a deterministic pathogen transport model for wetlands within a Bayesian statistical framework. Schijven et al. (2015) developed the probabilistic-deterministic microbial fate, transport and infection risk model QMRAcatch. This model was later applied by Derx et al. (2016) for evaluating the impact of human fecal pollution in a large riverine wetland. Daniels et al. (2014), Schijven et al. (2015), and Derx et al. (2016), however, did not account for the complex spatiotemporal variations of the flow and transport processes in wetlands.

The primary aim of this study was to develop a new integrative modeling approach supported by MST markers for quantifying the impact of human and animal, as well as, autochthonous and allochthonous fecal sources on the microbiological quality of an alluvial backwater channel considering safe drinking water production. The model should be able to account for the transient spatiotemporal variations of flow patterns and for the uncertainty of the source and transport variables. The secondary aim was to test the developed model at a Danube backwater area supported by MST markers and *E. coli* for quantifying the concentrations and loads of *Cryptosporidium* and *Giardia* (oo)cysts considering safe drinking water. These are important reference protozoa occurring ubiquitously in human and animal fecal sources (Stalder et al., 2011). *Cryptosporidium* and *Giardia* (oo)cysts are highly persistent in the environment and infectious at low dose (de Regnier et al., 1989; Boyer et al., 2009), making them suitable to address our research question. To meet our aims, we modified and extended QMRAcatch (v 1.1 python backwater). We defined event-driven scenarios of microbial transport into the backwater channel, (i) via the river entering the backwater during floods (allochthonous sources), (ii) via the resuspension from fecal deposits during flooding and inundation of the backwater area, and, (iii) via the release and runoff from fecal deposits during rainfall within the backwater area (autochthonous sources). These were assumed to be the most important transport pathways, and human wastewater, ruminants, wild boar (pigs) and birds were considered as the most important fecal pollution sources, based on the findings of Frick et al. (2020) in our study area. To validate this assumption, we used a comprehensive 6-year monthly monitoring dataset of human and animal MST markers and *E. coli*.

## Materials and Methods

### Study Area

**FIGURE 1:**
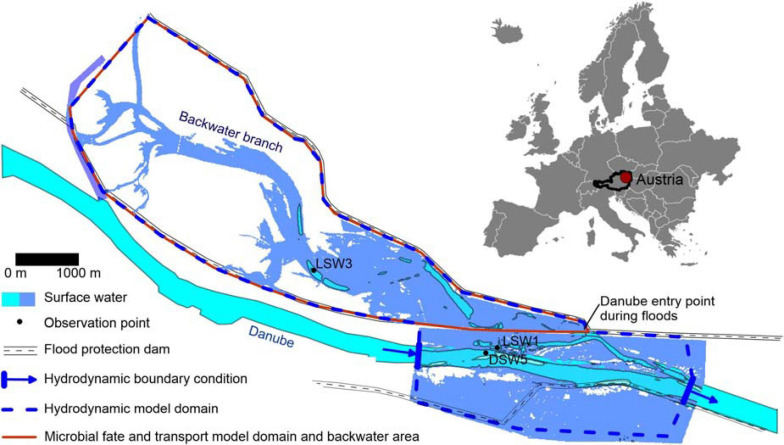
Study area with observation points DSW5 (Danube), LSW1 (affected by Danube discharge and inundation) and LSW 3 (affected by rainfall-release from fecal deposits) (Frick et al., 2020), QMRAcatch and hydrodynamic model domain; the surface water area simulated during the peak of the flood event in January 2011 is indicated in blue.

The investigated alluvial model backwater area is situated at the Danube at the downstream end of the city of Vienna, in Austria (Figure 1, see Frick et al., 2020 for a detailed description). The catchment upstream of Vienna is home to approximately 11 million inhabitants (Schreiber et al., 2005). Considering that 99% of the human population in the study area is connected to a WWTP (European Commission, 2018), urban wastewater is the main source of human pollution. Due to the regulation of the Danube, the backwater area has been almost completely disconnected from the main stream. The backwater presently consists of a channel network, whose main lateral branch has a surface connection with the Danube River at its lowermost end through a levee opening (‘entry point during floods’, Figure 1). Floods that enter the floodplain via the backflow connection move upstream along the main lateral branch, creating a distinct gradient in hydrologic connectivity in the various waterbodies with distance to the inflow (Reckendorfer et al., 2013). The backwater channel connects with the Danube River if the discharge exceeds 2200 m^3^/s (mean discharge: 1900 m^3^/s). Danube water enters the backwater channel and its lateral branches during the rising limb of flood events, causing inundation of parts of the surface area (Figure 1). When the flood peak is reached, the flow direction reverses, and the water flows back toward the Danube River.

The backwater area is 14 km^2^ in size, and represents an important water resource for drinking water supply for Vienna (Hein et al., 2006). There are five drinking water wells (riverbank filtrate) situated in the area. It is also part of a national park that plays a strategic role as a wilderness area and for recreation (Arnberger et al., 2009). There is no livestock in the considered backwater area, but there is a considerable population of wild animals, such as ruminants, wild boars and birds (Frühauf and Sabathy, 2006a,b; Parz-Gollner, 2006; Arnberger et al., 2009). Population sizes estimated for 2010 resulted in 180 red deer (*Cervus elaphus*), 44 roe deer (*Capreolus capreolus*), 17 fallow deer (*Dama dama*), 20 European mouflon (*Ovis orientalis musimon*) and 150 wild boars (*Sus scrofa*) (Government of the City of Vienna, Alexander Faltejsek, personal communication). Hunting is allowed in the area. The bird abundance is nearly 2500 at maximum in total (Frühauf and Sabathy, 2006a,b; Parz-Gollner, 2006; Schulze and Schütz, 2013). In addition, 600,000 visitors visit the national park area every year (Hinterberger et al., 2000). Bathing is prohibited at the considered backwater area, and visitors may only use selected paths for walking and cycling. Therefore, the possibility for human fecal input from visitors is considered to be low (Frick et al., 2018).

### Modeling Approach

**FIGURE 2:**
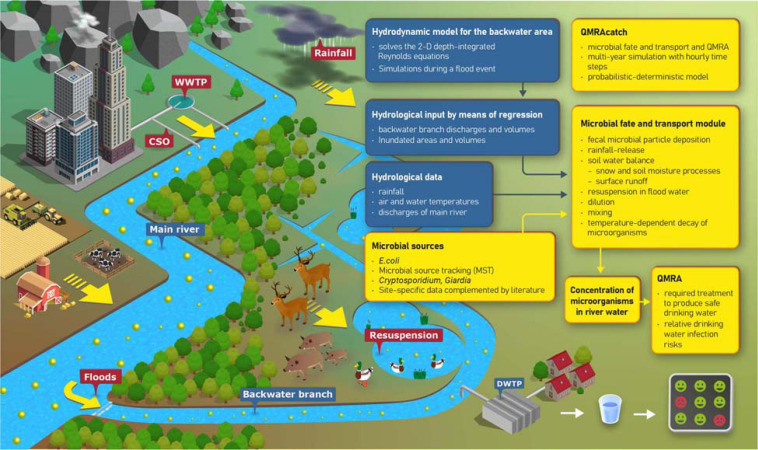
Microbial sources and transport processes in the alluvial backwater area (graphics). Components and data flows of the integrative modeling approach (flow chart).

For this paper, we used a modified and extended version of the probabilistic-deterministic microbial fate and transport and infection risk model QMRAcatch (Schijven et al., 2015; Demeter et al., 2021; Figure 2). We took a two-step modeling approach: (1) To test the assumption of the most relevant fecal sources and transport processes, we performed a source-targeted model validation using measured concentrations of host-associated MST markers and *E. coli* at the study site (Section “Validation of the Microbial Fate and Transport Model”). (2) To evaluate the importance of animal versus human, or autochthonous versus allochthonous sources in terms of potential health relevance, we then simulated various pollution scenarios (Section “Scenario Load and Infection Risk Assessment”). In these scenarios, we simulated the concentrations and loads of *Cryptosporidium* and *Giardia* in the backwater channel, and the required pathogen treatment reduction and daily drinking water infection risks relative to a health-based benchmark. The model domain of QMRAcatch encompasses the total backwater area, delimited by the Danube along the southern boundary and a flood protection dam on its northern end (Figure 1). Hydrological monitoring data together with 2-D hydrodynamic flow simulations during a flood event (Section “Hydrological and Hydrodynamic Flow Situation”) as well as measured microbial source data complemented with literature data (Section “Microbiological Source Characterization”) served as input to the model.

### QMRAcatch

#### Model Overview

The probabilistic-deterministic microbial fate and transport and infection risk model QMRAcatch (Schijven et al., 2015) was extended for this study and coded as open source (v1.1 Python backwater). QMRAcatch was used to simulate the microbial concentrations of the backwater channel (Sections “Microbial Fate and Transport Module” to “Microbiological Source Characterization”) and the daily drinking water infection risks after further treatment of the source water (Section “Quantitative Microbial Risk Assessment Module”). The model comprises the functionality of the version QMRAcatch 1.0 Python (Demeter et al., 2021) with the following extensions:

• Simulation time steps of 1 h in contrast to 1 day in the previous version.
• The concentration of MST markers, fecal indicators and pathogens in the main river (Danube) water is described by a statistical distribution according to the observed data set.
• Microbial particle release from animal fecal deposits is described as a function of precipitation and elapsed time since the start of precipitation according to Bradford and Schijven (2002).
• Hourly discharges and volumes of the backwater channel, flooded areas and floodplain volumes are additional input variables.
• Model equations were included to calculate surface runoff based on transient evaporation and soil moisture processes using air temperature and rainfall as input variables.
• Microbial decay in animal feces is described based on a uniformly distributed first order rate coefficient μ_*f*_. In the earlier version, the decay rates in water and feces were not differentiated.
• The prevalence of the reference pathogens *Cryptosporidium* and *Giardia* in animal waste is described by a mixture of beta distributions from reported studies following the methodology of Dorner et al. (2004).
• Data on times of consumption and consumed volumes of unboiled drinking water per person per day by the Dutch National Food Consumption Survey 2007–2010 (DNFCS) (Van Rossum et al., 2011) are used to calculate the daily cumulative dose and the daily drinking water infection risks.

#### Microbial Fate and Transport Module

Microbial contamination of the backwater channel occurs from the following three reservoirs: Danube, non-flooded and flooded area. Microbial particles are transported via inflows of Danube water during floods, by resuspension from animal deposits in inundated areas, and during rainfall causing release and runoff from animal fecal deposits in non-flooded areas.

##### Transport via the Danube entering the backwater during floods

**FIGURE 4:**
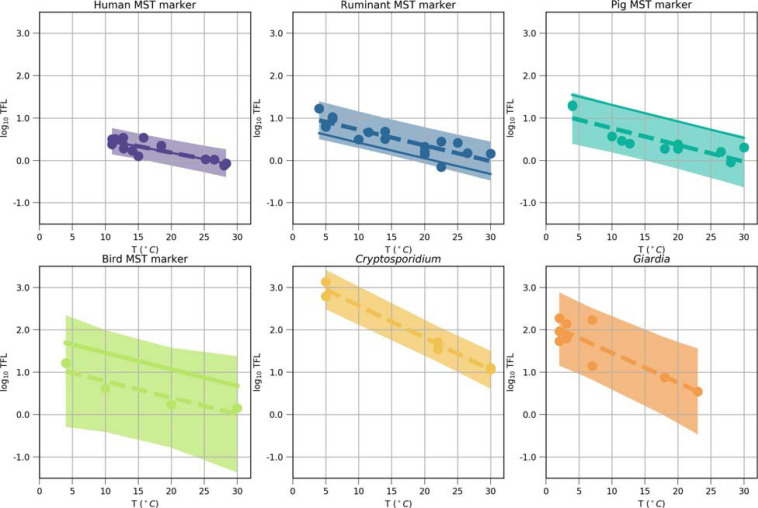
Inactivation of the MST markers, *Cryptosporidium*, and *Giardia* after model optimization (solid lines) and as reported in experimental studies (dots, Supplementary Table 4), plotted as time to first log_10_ reduction (TFL, days) values log_10_-transformed (log_10_ (TFL) in function of the temperature. Ordinary-least-square regressions (dashed lines) were fitted to the literature values, shown with their 95% prediction intervals (shaded). The intercept and the slope of the solid lines were the result of the model optimization. These values were used as model input parameters a_0_ and a_1_ in Equation 2 (Table 1).

Microorganisms carried by Danube water are subjected to mixing with the backwaters and temperature-dependent decay. Assuming steady-state conditions and complete mixing on each hour, the analytical solution for the microbial concentration in the backwater channel, *C*_*r→bw*_ [particles/L] at time step t is (Schijven et al., 2015):


(1) $$\begin{array}{l} C_{r\to bw}(t)=\frac{Q_{bw}}{Q_{bw}+{\text{μ}}_{w}(T_{bw})V_{bw}}C_{r}+(C_{r\to bw}(t-1)\\ -\frac{Q_{bw}}{Q_{bw}+{\text{μ}}_{w}(T_{bw})V_{bw}}C_{r})exp(-\frac{Q_{bw}+{\text{μ}}_{w}(T_{bw})V_{bw}}{V_{bw}}t) \end{array}$$

where *C*_*r*_ [particles/L] is the microbial particle concentration of the Danube river, *Q*_*bw*_ and V_*bw*_ are the discharge and volume of the backwater channel (Section “Hydrological and Hydrodynamic Flow Situation,” Supplementary Table 2). The degree of reduction of microorganisms during the transport depends on the travel time or flow rate. Decay during transport is described as a first order reaction, where the decay rate in water (μ_*w*_ [1/d]) is a function of the water temperature (T_*bw*_ [°C]):


(2) $$\mu_{w}\,\left(t\right)=\frac{l\,n\,10}{10^{a_{0}+a_{1}\,T_{b\,w}}}$$

where a_0_ [log_10_ day] and a_1_ [log_10_ day/°C] are microorganism-specific decay rate parameters (Bertrand et al., 2012). We conducted an extensive literature review on the microorganism-specific decay rates in water and adjusted the values within prediction intervals (Supplementary Table 4, Figure 4). The rates implicitly included additional removal or regrowth processes, or effects of other environmental factors, such as temperature, pH, TOC (van Elsas et al., 2011; Ahmed et al., 2019).

**TABLE 1:** Microorganism-specific model input parameters in QMRAcatch.

Parameter	Dimension	Details	Distribution type	Microorganism	Value	References
Concentrations of feces C_*f*_ (mean, 95^*th*^ percentile)	N/g	Deer sources	Gamma	*E. coli*	(3, 16) × 10^7^	Farnleitner et al., 2010
				Human MST	(3, 11) × 10^3^	Farnleitner et al., 2014
				Ruminant MST	(2, 6) × 10^9^	Farnleitner et al., 2014
				Pig MST	(3, 20) × 10^3^	Farnleitner et al., 2014
				Duck MST	(7, 52) × 10^3^	Farnleitner et al., 2014
				*C. bovis + C. ryanae*	103, 225	Garcia-Presedo et al., 2013
				*Giardia duodenalis A-II*	89, 320	Garcia-Presedo et al., 2013
		Pig sources	Gamma	*E. coli*	(0.04, 2.5) × 10^8^	Frick et al., 2018
				Human MST	(7, 33) × 10^3^	Farnleitner et al., 2014
				Ruminant MST	0, 0	Farnleitner et al., 2014
				Pig MST	(2, 10) × 10^10^	Farnleitner et al., 2014
				Duck MST	0, 0	Farnleitner et al., 2014
				*Cryptosporidium*	70, 133	Castro-Hermida et al., 2011
				*Giardia*	8, 10	Castro-Hermida et al., 2011
		Bird sources	Gamma	*E. coli*	(0.001, 3.2) × 10^8^	Frick et al., 2018
				Human MST	(2, 6) × 10^3^	Farnleitner et al., 2014
				Ruminant MST	(1, 9) × 10^3^	Farnleitner et al., 2014
				Pig MST	(8, 30) × 10^4^	Farnleitner et al., 2014
				Duck MST	(1.3, 4.5) × 10^7^	Farnleitner et al., 2014
				*Cryptosporidium parvum*	288, 686	for Canada migratory geese (Graczyk et al., 1998)
				*Giardia*	405, 786	for Canada migratory geese (Graczyk et al., 1998)
**a_0_, a_1_**	log_10_ day, log_10_ day/ °C	Parameters to describe first order decay in water as function of water temperature	Constant	Human MST marker	0.78, −0.03	This study (best fit)
				Ruminant MST marker	0.79, −0.037	This study (best fit)
				Pig MST marker	1.7, −0.039	This study (best fit)
				Bird MST marker	1.85, −0.039	This study (best fit)
				*E. coli*	1.04, −0.017	Franz et al., 2014
				*Cryptosporidium*	3.3, −0.076	Ives et al., 2007
				*Giardia*	2.16, −0.07	de Regnier et al., 1989
**μ_*f*_ minimum, maximum**	1/d	first order decay in feces in all animal sources based on reported values in bovine feces	Uniform	Ruminant MST marker	−0.13, −0.11	Oladeinde et al., 2014
				*E. coli*	−0.3, +0.1	Oladeinde et al., 2014
				*Cryptosporidium*	−0.05, −0.03	Olson et al., 1999
				*Giardia*	−0.38, −0.11	Olson et al., 1999
**a, β**	1/mm, -	release parameters		All microorganisms	0.1, 2.0	Bradford and Schijven, 2002; Guber et al., 2015
**Dose-response model parameters**						
**α, β**	-	hypergeometric		*Cryptosporidium*	0.3, 1.1	Schijven et al., 2011, 2015
**r**	-	exponential		*Giardia*	0.02	Regli et al., 1991

*Decay rate coefficients in fresh water and in feces (Equations 2 and 9), release parameters (Equation 4), and dose-response parameters α and β (Equation 13).*

##### Microbial particle deposition

Microbial particle loadings from animals were determined using the method described by Dorner et al. (2004) and Sterk et al. (2016). For pathogens, the fraction of animals infected by *Cryptosporidium* or *Giardia* was derived by random sampling from several (equally weighted) beta-distributions, describing the probability that an animal is positive (prev):


(3) p⁢r⁢e⁢v∼β⁢(α,β)

Parameters a and b for each of the b-distributions are based upon prevalence studies (see Supplementary Table 1). In the selection process, studies were prioritized based on their recentness, number of samples and location. Studies conducted in temperate, high-income regions were given priority.

For each infected animal, the microbial particle numbers shed per hour were determined by multiplying the mass of feces per dropping m_*f*_ (kg, normally distributed), the number of droppings per hour per animal (Poisson distributed) and the microbial concentration (Tables 1, 2). The microbial concentration in feces is described by a gamma distribution based on mean and 95th percentile values reported in the literature (Table 1). Fecal deposition only takes place in the non-flooded area of the floodplain.

**TABLE 2:** Input parameter settings for the animal sources in QMRAcatch.

Parameter	Unit	Value	References	Value	References	Value	References

		*Deer*		*Wild boar*		*Birds*	
Population size	N	240	Vierheilig et al., 2013; Böhm, 2016	200	Vierheilig et al., 2013, MA 49, personal communication	2,500	Frühauf and Sabathy, 2006a,b; Parz-Gollner, 2006; Schulze and Schütz, 2013
Weight (Mean, 95%)	g	15,30	von Oheimb et al., 2005	10, 20	Schmidt et al., 2004	0.5, 1	Hahn et al., 2007
Defecation rate	1/h/animal	0.63	von Oheimb et al., 2005	0.2	Schmidt et al., 2004	2.1	Hahn et al., 2007

##### Transport in non-flooded area

Rainfall-induced microbial particle release was modeled iteratively using the function for the release of *Cryptosporidium* and *Giardia* from dairy cattle manure of Bradford and Schijven (2002). The release rate is given by:


(4) $$\omega \,\left(t\right)=a\,P\,\left(t\right)\,{\left[1+a\,\beta \,P\,\left(t\right)\,t_{r\,a\,i\,n}\,\left(t\right)\right]}^{-\left(1+\frac{1}{\beta }\right)}$$

according to Guber et al. (2015), where P (mm/h) is the amount of rainfall at time step t, t_*rain*_ is the time passed since the start of the rainfall event, a (1/mm) controls the initial release rate, and β (–) determines the shape of the release curve (Table 1). Release from the deposits occurs in the non-flooded area (A_*dep*_ [m^2^]). First, the available number of microbial particles in animal fecal deposits N_*deptot*_ is determined from the newly deposited numbers (N_*dep*_) plus the residual deposits of the previous time step (N_*depres*_, Equation 9):


(5) $$N_{d\,e\,p\,t\,o\,t}\,\left(t\right)=N_{d\,e\,p}\,\left(t\right)+N_{d\,e\,p\,r\,e\,s}\,\left(t-1\right)\,△\,A_{d\,e\,p}\,\left(t\right)$$

where


(6) △A(t)d⁢e⁢p=Min[1,A(t)d⁢e⁢p/A(t-1)d⁢e⁢p].

If the size of the non-flooded area decreases, △A_*dep*_ is smaller than one, otherwise it is one. The number of microbial particles that are released in the non-flooded area are then calculated from the total number of microbial particles in animal fecal deposits (N_*dep*__*tot*_):


(7) $$N_{r\,e\,l,n\,o\,n\,f\,l\,o\,o\,d\,e\,d}\,\left(t\right)=\left\{ \begin{array}{c} P_{Q}=0\to 0\\ P_{Q}>0\to \omega \,N_{d\,e\,p\,t\,o\,t}\,\left(t\right)\cdot \text{exp}\,\left[\mu_{w}\,\left(t\right)\right] \end{array}\right.$$

where μ_*w*_ is determined according to Equation 2. We calculated the surface water runoff volume P_*Q*_ [m^3^/h] as function of precipitation (P, including rain water and snow melt), evaporation and soil moisture processes according to Blöschl et al. (2008), Equations 1 – 4. To calculate the changes in soil moisture we used Equations 5, 7 and 8 given by Blöschl et al. (2008), with parameter settings according to Demeter et al. (2021) for the study site (Supplementary Table 3).

##### Transport in flooded area

Animal fecal deposits are completely resuspended in floodwater. The number of resuspended microbial particles, N_*rel,flooded*_, is:


(8) $$N_{r\,e\,l,f\,l\,o\,o\,d\,e\,d}\,\left(t\right)=\left\{ \begin{array}{c} △\,A_{d\,e\,p}=1\to 0\\ △A_{d\,e\,p}<1\to N_{d\,e\,p\,r\,e\,s}\cdot \left(1-△A_{d\,e\,p}\right)\cdot \\ \text{exp}\,\left[\mu_{w}\,\left(t\right)\right] \end{array}\right.$$

where μ_*w*_ and △A_*dep*_ are determined according to Equations (2) and (6). The number of released microbial particles is then subtracted from the total deposited numbers and reduced by first-order decay in feces, μ_*f*_ [1/h]:


(9) $$N_{d\,e\,p\,r\,e\,s}\,\left(t\right)=\left[N_{d\,e\,p\,t\,o\,t}\,\left(t\right)-\omega \,\left(t\right)\,N_{d\,e\,p\,t\,o\,t}\,\left(t\right)\right]\cdot \text{exp}\,\left[\mu_{f}\,\left(t\right)\right]$$

For μ_*f*_, we took ranges of reported values for bovine feces as the boundaries of a uniform distribution according to Wu et al. (2020). This distribution was assumed to evenly represent the varying μ_*f*_ values with environmental conditions and time (Table 1). The number of microbial particles running off to the backwater channel in non-flooded and flooded areas are then added:


(10) $$N_{v\,r\,o}\,\left(t\right)=N_{r\,e\,l,n\,o\,n\,f\,l\,o\,o\,d\,e\,d}\,\left(t\right)+N_{r\,e\,l,f\,l\,o\,o\,d\,e\,d}\,\left(t\right)$$

The numbers of microbial particles from each animal group are summed. The microbial particle concentrations in the backwater channel are then calculated:


(11) $$C_{b\,w}\,\left(t\right)=\left\{ \begin{array}{c} V_{f\,l}\,\left(t\right)+P_{Q}\,\left(t\right)>0\to \frac{C_{r\to b\,w}\,\left(t\right)\cdot V_{f\,l}\,\left(t\right)+N_{v\,r\,o}\,\left(t\right)}{V_{f\,l}\,\left(t\right)+P_{Q}\,\left(t\right)}\\ V_{f\,l}\,\left(t\right)+P_{Q}\,\left(t\right)=0\to C_{r\to b\,w}\,\left(t\right) \end{array}\right.$$

where *V*_*fl*_ [m^3^] is determined by means of regression (Section “Hydrological and Hydrodynamic Flow Situation,” Supplementary Table 2).

#### Hydrological and Hydrodynamic Flow Situation

**FIGURE 3:**
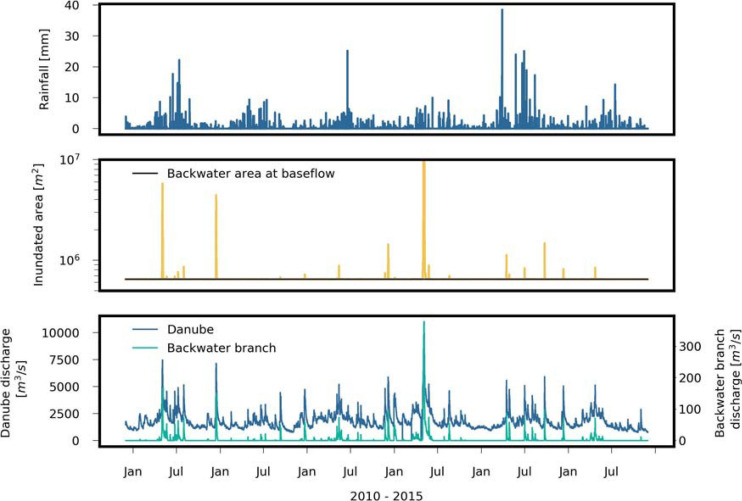
Observed hourly rainfall and Danube discharge, and, simulated hourly discharge of the backwater channel and inundated area by means of regression (Supplementary Table 2) during the investigation period.

We selected the years 2010–2015 as study period. The lowest Danube discharges in this period occurred in 2011 (Q_95_: 2400 m^3^/s), and the highest discharges in 2013 (Q_95_: 4100 m^3^/s, Figure 3). Hourly discharge data of the Danube were available at gauge Wildungsmauer, which is located 12 km downstream of the study site. The annual precipitation was 452 mm in 2011 and 659 mm in 2013. Hourly precipitation data (mm/h) and air temperature was available at station Groß Enzersdorf, located at 6 km distance from the Danube along the upstream model boundary, Figure 1), the latter ranging from −20°C to 38°C (mean: 11.2°C, standard deviation: 8.9°C). The water temperature of the Danube (gauge Greifenstein, 41 km upstream of the study area) ranged from 0 to 23°C (mean: 11.2°C, standard deviation: 5.9°C).

A 2-D hydrodynamic surface water model (CCHE2D Version 2.0, National Center for Computational Hydroscience and Engineering, University of Mississippi) was used to simulate the flow velocities and water levels of the backwater channel on an hourly basis during a flood event in January 2011 with an approximate 10-year return period. The model is described in detail by Gabriel et al. (2014) and Frick et al. (2020). In short, the model solves the two-dimensional formulation of the shallow water equations and uses depth integrated Reynolds equations. For temporal discretization, the implicit first order Euler’s method was implemented and was able to simulate subcritical and supercritical flow conditions. The model domain covered an area of approximately 22 km^2^ (Figure 1). The spatial distribution of the Manning roughness values were based on a detailed land use and vegetation classification. During model calibration the Manning roughness values were fine-adjusted to floods of the River Danube during August 2008 and June 2009, ranging from 0.024 to 0.125 s⋅m^–0.3^ within the model domain. The Nash and Sutcliffe coefficient of runoff model efficiency (Nash and Sutcliffe, 1970) at several water level gauges along the backwater branch from the inlet point of the Danube to LSW 3 (Figure 1) ranged from 0.92 to 0.98 for both calibration periods, and from 0.93 to 0.96 for the validation period during the flood event in January 2011.

The transient water quantities were determined by means of polynomial regression based on the hydrodynamic flow simulations during the rising limb of the flood event (Supplementary Table 2). The following variables were calculated and served as input to the microbial fate and transport model: hourly discharges and volumes of the backwater channel, and hourly volumes and areas of inundation (see Supplementary Section “Detailed Model Information” for more details). The shortest and longest period when the Danube discharged into the backwater channel were 16 days in 2011, and 91 days in 2013, respectively.

#### Microbiological Source Characterization

##### Microbial analyses of surface water

Surface water samples were collected monthly from 2010 to 2015. Samples were collected from one point at the Danube (DSW5), and from points LSW 1 and 3 along the backwater channel (Figure 1). The sampling location LSW1 is situated in a lateral branch outside the flood-protected area delineated by the dam and therefore represents waterbodies with high connectivity to the Danube River. The location LSW3 represents waterbodies along the main backwater channel, with an average water depth of ca. 170 cm. The MST markers were quantified in 500–600 mL water samples using quantitative PCR. The human marker HF183/BacR287 (Green et al., 2014), the ruminant marker BacR (Reischer et al., 2006), the pig marker Pig2Bac (Mieszkin et al., 2009), and the bird marker DuckBac (Kobayashi et al., 2013) were selected and applied as described previously (Kirschner et al., 2017). As a robust approximation for the SLOD (sample limit of detection), which can only be determined by elaborate spiking processes to determine sample processing efficiencies on a sample-to-sample basis (filtration- and extraction efficiencies with representative MST mock communities), we applied the established threshold of detection (TOD) concept for MST field applications (Reischer et al., 2007, 2008). The filtration volume (200 - 300 mL), the use of 2.5 ml of diluted DNA extract in qPCR and the minimal amount of detectable targets per PCR reaction defines the detection threshold (Reischer et al., 2006, 2007). The quantitative microbial source tracking results were then expressed as marker equivalents per L (ME/L) to account for potential extraction losses (Reischer et al., 2007, 2008). The TOD covers sampling and sample processing information and also the efficiency of qPCR analysis. The mean TODs during the calibration and validation periods were 564 and 382 ME/L for the human, 419 and 327 ME/L for the ruminant, 490 and 337 ME/L for the pig, and 419 and 327 ME/L for the bird MST marker. The samples were analyzed for *E. coli* according to ISO 16649-1 (ISO, 2001) with a limit of detection (LOD) of 1 CFU/100 mL. Additional surface water samples were collected monthly at the Danube 23 km upstream of DSW 5 from June 2018 to August 2020 and analyzed for *Giardia* and *Cryptosporidium* (oo)cysts. *Giardia* spp. and *Cryptosporidium* spp. (oo)cysts were isolated from 10-L water samples, using an adaptation of the flat membrane method described in ISO (2006). Parasites were recovered from the filters and further analyzed as described in Demeter et al. (2021) using 50 mL of 1M glycine pH 5.5 solution and centrifuged at 1,550 × g for 15 min. Pellets were resuspended in 2 mL of ultrapure water. One mL of the suspension was used for the immunomagnetic separation of the parasites using the Dynabeads GC Combo kit (Thermo Fisher, United Kingdom). Concentrates were stained with the EasyStain kit (BTF Pty. Ltd., Biomerieux, Australia) and quantified as described by Stevenson et al. (2015). The LOD of *Giardia* and *Cryptosporidium* in surface waters was 0.4 (oo)cysts/L.

##### Microbial concentrations in animal feces

Samples of fecal matter of ruminants (hunted herbivores), wild boar, avian fecal matter from great cormorant (*Phalacrocorax carbo*), wild duck (*Anas platyrhynchos*) and other *Anatidae*, common tern (*Sterna hirundo*), and *Charadriiformes* were previously collected and analyzed for MST markers and *E. coli* in the study area (Vierheilig et al., 2013; Farnleitner et al., 2014; Frick et al., 2018). Marker concentrations associated with human (*n* = 19), ruminant (*n* = 20), porcine (*n* = 18), and bird fecal pollution (*n* = 11) from these samples were determined via qPCR (Farnleitner et al., 2014; Table 1). For *Cryptosporidium* and *Giardia*, reported values were used (Table 1).

##### Data analysis

The fecal indicator and pathogen concentrations in the Danube, C_*r*_, used in Equation 1, were described by selected statistical distributions (Table 3). The parameters of the distributions were obtained from fits to the observed dataset at point DSW 5 (Figure 1). We performed Kruskal–Wallis tests for the selection of the distribution types (*p* > 0.05, Table 3). During all model simulations, random values were drawn from the distributions for each time step and Monte Carlo run. A substantial fraction of the measured microbial concentrations were left-censored values (i.e., 20, 60, 76, 30% for the human, ruminant, pig, and bird MST markers, and, 32 and 39 % for *Giardia* and *Cryptosporidium*), meaning that the concentration was known only to be lower than the LOD (*Giardia* and *Cryptosporidium*) or the TOD (MST markers). Non-detects (ND) were replaced by half of the TOD in case of the MST markers and by half of the LOD in case of *Cryptosporidium* and *Giardia*. This continues to be the most common procedure within the disciplines of environmental sciences to deal with non-detects (Helsel, 2006). Concerning the MST data, we used these in the calibration process. Since we treated both simulated and observed values the same way (i.e., observed non-detects and simulated values < TOD were both replaced by TOD/2), the chosen performance metrics are not affected, leading to the best calibration possible. Concerning the *Giardia* and *Cryptosporidium* data, we compared different levels for the substitution, i.e., substitution by zero, LOD/2 and LOD. It was shown that the choice of the level did not affect the results of the Kruskal–Wallis tests (*p* > 0.05), so the method was justifiable in our case. For modeling the concentrations of *Giardia* and *Cryptosporidium* in the backwater branch and the QMRA, we merely used the data in the Danube as boundary condition. We did not use the data in the backwater river for this purpose, where the concentrations would be lower than the LOD in most cases. For the data analysis, Python 3.7 and Scipy package 1.3.1 were used.

**TABLE 3:** Observed values and descriptive statistics for the microbial concentrations of the Danube from 2010 to 2015 (C_*r*_ in Equation 1) and the Kruskal–Wallis test results showing that the simulated and observed values were not significantly different (*p* ≥ 0.05).

Parameter	Observed dataset detected/n	Observed median (minimum, maximum) [particles/L]	Distribution	Descriptive statistical parameter values gamma distribution: shape/location/scale; normal distribution: mean/standard deviation	Kruskal–Wallis *p*	Comment
Human MST marker	50/62	5.39 × 10^3^ (60, 1.41 × 10^6^)	Gamma	0.23 / 58 / 80,000	0.05	After model optimization based on OF (Equation 14)
Ruminant MST marker	21/60	250 (60, 4.54 × 10^4^)	Gamma	0.23 / 58.5 / 10,766	0.14	
Pig MST marker	14/60	220 (60, 1.58 × 10^5^)	Gamma	0.25 / 58.5 / 3,700	0.11	
Bird MST marker	16/23	1.21 × 10^4^ (60, 2.19 × 10^5^)	Gamma	0.45 / 58 / 20,000	0.05	
*E. coli*	64/64	570 (20, 4.9 × 10^4^)	Gamma	0.19 / 100 / 27,000	0.33	
*Giardia*	21/31	0.8 (0.2, 4.4)	Normal	1.39 / 1.17	0.45	stats fit function in Python 3.7
*Cryptosporidium*	19/31	0.8 (0.2, 6.44)	Normal	1.29 / 1.42	0.40	

*Non-detects were replaced by half of the TOD (MST markers) or half of the LOD (*E. coli*, *Giardia*, and *Cryptosporidium*).*

#### Quantitative Microbial Risk Assessment Module

Exposure to the pathogens is given as the dose D [L/d], the number of ingested pathogens per person per day. For calculating D, the Monte Carlo samples of pathogen concentrations in the backwater branch (C_*bw*_ [particles/L]), recovery (R, [-]), pathogen treatment reduction (log reduction value, LRV), and consumption data (V, [L]) are multiplied according to Equation 12. Data on times of consumption and consumed volumes of unboiled drinking water per person (V_*i*_ in equation 12) during a day were available from the Dutch National Food Consumption Survey 2007–2010 (DNFCS) (Van Rossum et al., 2011). The cumulative dose per person per day is:


(12) $$D=\sum_{i=1}^{d}\left(C_{b\,w,i}\times \frac{1}{R}\times 10^{L\,R\,V}\times V_{i}\right)$$

where i denotes the hourly time step. Recovery rates were determined in the laboratory, resulting in mean values of 0.65 for *Giardia* (standard deviation: 0.28, *n* = 17) and 0.53 for *Cryptosporidium* (standard deviation: 0.27, *n* = 8). Beta distributions were fitted to the recovery data (α: 0.87, β: 0.53 for *Cryptosporidium* and α: 1.03, β: 0.44 for *Giardia*). Daily probabilities of infection for *Cryptosporidium* can be estimated using a hypergeometric dose–response relation (Teunis and Havelaar, 2000):


(13) Pi⁢n⁢f=1-F11⁢(α,α+β,D)

where α and β are infectivity parameters that are pathogen-specific and _1_F_1_ is the confluent hypergeometric function. The dose-response model parameters for *Cryptosporidium* and *Giardia* were taken from the literature (Table 1). As daily health based target (hbt), 1⋅10^−6^ infections/person/d was adopted in this study (Signor and Ashbolt, 2009). LRV was estimated iteratively until the criterion *P*_*inf*_ ≤ hbt according to Equation (13) was fulfilled for both the mean and 95^*th*^ percentile values of P_*inf*_.

### Validation of the Microbial Fate and Transport Model

To prove that the model captured the most relevant fecal sources and transport processes, we evaluated the model performance based on monthly measured concentrations of the human and animal MST markers and *E. coli* during 2010–2015. We selected the observation dates, when Danube discharged into the wetland area, or when rainfall occurred. The mean absolute error (MAE) was used as a performance metric (Willmott and Matsuura, 2005; Demeter et al., 2021). Log_10_ transformed concentrations were used in the MAE computations because microorganisms typically follow a lognormal distribution and the use of logarithms minimizes the influence of outliers present in the data (Hong et al., 2018; Demeter et al., 2021). The Kruskal–Wallis test was used for the distribution comparisons of the simulated and observed datasets in the backwater channel and the p-value of the Kruskal–Wallis statistic was a metric of model performance. In order to ensure an optimum model performance, the optimization parameters were adjusted to minimize the objective function (OF) (Demeter et al., 2021).


(14) O⁢F=M⁢A⁢E+(1-p)

Non-detects (ND) in the observed dataset and simulated values below that level were set to the half of the threshold of detection (for the MST markers) in the calculations. The optimization parameters were the distribution parameters describing the microbial concentrations in the Danube and the microorganism-specific decay rate parameters (C_*r*_ in Equation 1, a_0_ and a_1_ in Equation 2). The former were adjusted while ensuring that the Krukal-Wallis p-value was greater than 0.05 (Table 3). Decay rates of the MST markers were collected from a literature survey and summarized in Supplementary Table 4. An ordinary least square method was used to fit the time-to-first-log (TFL) as a function of water temperature (dashed lines in Figure 4), using the Python 3.7 package statsmodels (0.10.1). During the adjustment of intercept a_0_ (used in Equation 2, solid lines in Figure 4), it was ensured that the decay as function of temperature obtained lay within the prediction interval of the ordinary least square regressions (Figure 4, shaded area). The Bradford-Schijven release parameter a and β were kept the same for all animal sources, as their effects on the simulated concentrations was small in comparison with the optimization parameters (Table 1). We performed a stepwise, source-targeted model optimization of QMRAcatch:

• The model was validated, considering individual, presumably important fecal sources using measured concentrations of the respective MST markers. We simulated concentrations of the human MST marker in the backwater channel and compared them with the measured dataset on days when the Danube discharged into the backwater channel, i.e., during floods. We selected the data during days when the Danube discharged into the backwater channel (Section “Study Area,” Figure 1). The distribution parameters describing the human-associated MST marker concentrations in the Danube and the decay rate coefficient a_0_ were adjusted to minimize OF (Equation 14). The same procedure was applied consecutively for the ruminant, pig, and bird associated MST markers, except that animal fecal deposits were additionally considered as microbiological sources (Table 1). As for the human MST marker, we used the data collected during floods. In addition, we used data collected on days, when the backwater area was partially inundated or when it was raining (Section “Study Area,” Figure 1). The observation sites were selected based on the findings of Frick et al. (2020) who conducted a comprehensive analysis of the spatial distribution of human and animal fecal pollution in the study area. To validate the model during floods, we selected the site, which was influenced by floods (LSW 1). To validate the model during days of rainfall or inundation of the area, we used the site, which was impacted by wildlife (LSW 3, Figure 1).
• In the second step, the model was validated using measured concentrations of *E. coli*. The same procedure was applied as for the MST markers, except that we considered all fecal sources combined and the decay rate coefficients were not adjusted but taken from the literature (Table 1).

### Scenario Load and Infection Risk Assessment

We defined the following event-driven scenarios for quantifying the effects of fecal sources on the microbiological quality of the backwater channel considering safe drinking water:

• As allochthonous source, we considered *Cryptosporidium* and *Giardia* transport via Danube discharges into the backwater channel (scenario FLOODS).
• As autochthonous sources, we considered the resuspension of *Cryptosporidium* and *Giardia* from fecal deposits in inundated areas (scenario RESUSP), and the rainfall-release and runoff of *Cryptosporidium* and *Giardia* from fecal deposits (scenario RAIN).
• All of the above scenarios were considered simultaneously (scenario all combined).

Using QMRAcatch, we simulated the concentrations and loads of *Cryptosporidium* and *Giardia* in the backwater channel, and the drinking water infection risks relative to a health based target. We considered different hydrological conditions and fecal sources in the scenarios, as indicated in Table 4. To simulate no connection of Danube and backwater (Table 4), the Danube discharge was set to the mean flow rate, consequently there was no discharge into the backwater channel. All other parameter settings were taken from Tables 1–3 and Supplementary Material.

**TABLE 4:** Fecal sources and hydrological conditions in the event-driven scenarios investigating (i) the wastewater-impacted river water entering the backwater during floods (FLOODS), (ii) the resuspension of pathogens from fecal deposits in inundated areas (RESUSP), and (iii) the pathogen release and runoff from fecal deposits (RAIN), and (iv) all combined.

	Wastewater impacted river water (FLOODS)	Resuspension of pathogens from fecal deposits in inundated areas (RESUSP)	Pathogen release and runoff from fecal deposits (RAIN)	All combined
**Hydrological conditions**				
Rain	–	–	+	+
Connection between Danube and backwater	+	+	–	+
**Fecal pollution sources**				
River	+	–	–	+
Ruminants	–	+	+	+
Wild boar	–	+	+	+
Birds	–	+	+	+

*+ taken into account, – not applied.*

## Results

In order to test if we considered the most relevant fecal sources and transport pathways, we tested the applicability of the human and selected animal MST markers (Section “Applicability of the MST Marker Specificity for Modeling”), and validated the model based on measured concentrations of these MST markers and *E. coli* in the backwater channel (Section “Performance of the Microbial Fate and Transport Model”). We then simulated the *Giardia* and *Cryptosporidium* concentrations, loads and drinking water infection risks relative to a health-based benchmark for the defined fecal pollution scenarios and reference pathogens (Section “Contribution of Fecal Sources to the Reference Pathogen Impact on the Backwater Resource Considering Safe Drinking Water Production).

### Applicability of the MST Marker Specificity for Modeling

The selected human, ruminant, pig and bird MST marker assays (Section “Microbiological Source Characterization”) are primarily associated with their respective target sources. However, low numbers may also occur in the non-target pollution sources. According to the analysis of fecal samples, the reported MST marker concentrations in the non-target pollution sources were more than five orders of magnitude lower than those in the target pollution sources (Table 1). Nevertheless, the impact on false-positive MST marker detection rates in the backwater area may become significant, when a large non-target animal population is the source. To evaluate the applicability of the MST markers, we investigated the impact on the simulated concentrations from non-target animal sources in the floodplain river with QMRAcatch. The concentrations of each MST marker in the backwater channel were described by a gamma distribution based on the reported mean and 95^*th*^ percentile fecal source concentrations according to Table 1, and considering (i) both target (i.e., correct positive detections) and non-target host groups (i.e., false positive detections) and (ii) only the target group. The parameter settings were used according to Tables 1–3 in the simulations.

For each measured MST marker, the simulated mean and 95^*th*^ percentile concentrations in the backwater branch during the simulation period were compared for the two cases. For all MST markers, the simulated concentrations considering both target and non-target groups differed by 0 - 5 % from the simulated concentrations considering only the target group. This means that at least 95 % of the simulated concentrations in the floodplain river were associated with the target pollution sources in the catchment. The animal and human associated qPCR assays and the measured concentrations at our study site were thus considered to be useful for a source–targeted evaluation of the microbial fate and transport model.

### Performance of the Microbial Fate and Transport Model

**FIGURE 5:**
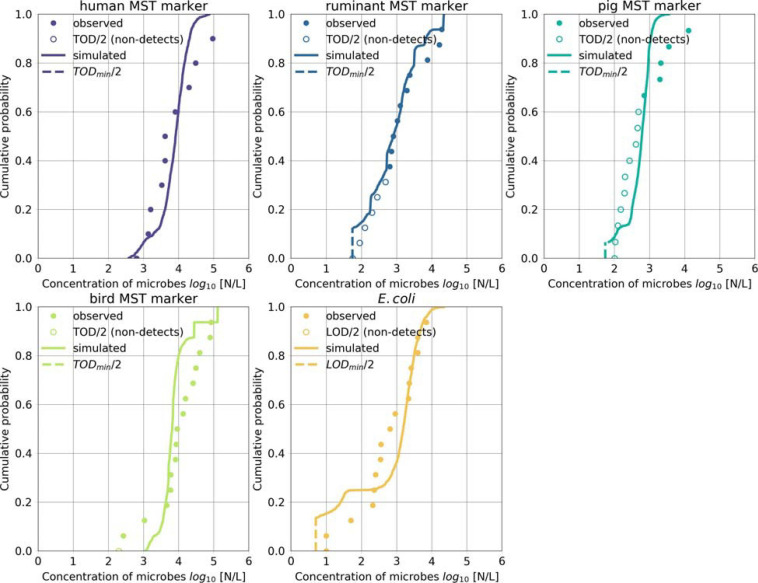
Microbial fate and transport model performance. Simulated and observed concentrations of the host-associated MST markers and of *E. coli* during days when Danube water discharged into the backwater branch or on rainy days. Non-detects were replaced by half of the TOD (MST markers) or LOD (*E. coli*). Simulated values below the LOD or the lowest TOD (TOD_*min*_) were set to half of the LOD or the TOD_*min*_.

**FIGURE 6:**
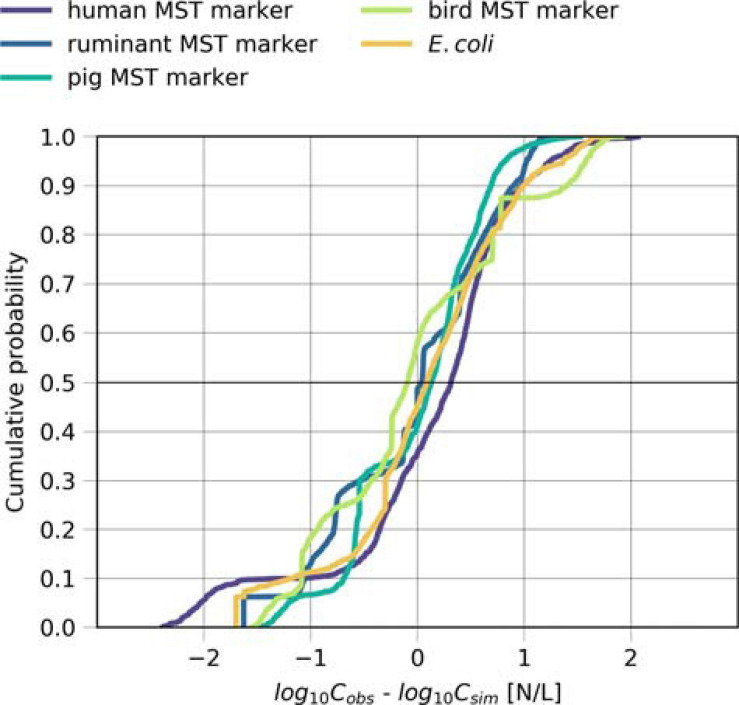
Model performance for the source-targeted microbial fate and transport. Cumulative distribution plot of the difference between the simulated and measured concentrations (log_10_-transformed) of the human, ruminant, pig and bird-associated MST markers and *E. coli* during days when Danube water discharged into the backwater branch or on rainy days.

We validated the model based on measured concentrations of human-, ruminant-, pig-, bird-associated MST marker and *E. coli* in the backwater branch. The model validation resulted in mean absolute errors ranging from 0.5 to 0.7 log_10_ for the MST markers and *E. coli* (Table 5). The objective function values ranged from 0.7 to 1.5 (OF in Equation 14). The cumulative distribution plot of the simulated and observed concentrations confirmed the general good agreement (Figure 5). The majority (70–90 %) of the simulated concentrations of all fecal indicators resulted in errors ranging from −1.0 to 1.0 log_10_ particles/L after model optimization (Figure 6).

**TABLE 5:** Model performance based on the observed microbial concentrations in 2010–2015 during days when the Danube discharged into the backwater branch and when rainfall occurred.

Parameter	Observed dataset	Observed median (minimum, maximum)	OF (Equation 14)	Mean absolute error	Kruskal–Wallis test
	Detected/n	[particles/L]		[log_10_ particles/L]	p
Human MST marker	9/10	4.1 × 10^3^ (640, 9.3 × 10^4^)	1.34	0.67	0.33
Ruminant MST marker	10/16	708 (56, 1.64 × 10^4^)	0.70	0.61	0.91
Pig MST marker	6/15	231 (65, 1.29 × 10^4^)	1.17	0.50	0.33
Bird MST marker	14/16	7.04 × 10^3^ (89, 8.37 × 10^4^)	1.53	0.69	0.16
*E. coli*	16/16	220 (10, 7.0 × 10^3^)	1.19	0.62	0.44

*Objective function values (OF), mean absolute error and Kruskal–Wallis *p*-values after comparison of the simulated and observed microbial concentrations in the backwater branch. Non-detects were replaced by half of the TOD (MST markers) or LOD (*E. coli*). Simulated values below the half of the LOD or the lowest TOD were set to the half of the LOD or lowest TOD.*

### Contribution of Fecal Sources to the Reference Pathogen Impact on the Backwater Resource Considering Safe Drinking Water Production

For the scenarios, we evaluated the simulated concentrations of *Cryptosporidium* and *Giardia* during time steps when

• The Danube discharged into the backwater branch (for the FLOODS scenario),
• Part of the backwater area was inundated (for the RESUSP scenario),
• Rainfall occurred (for the RAIN scenario), and
• All of the above combined.

**FIGURE 7:**
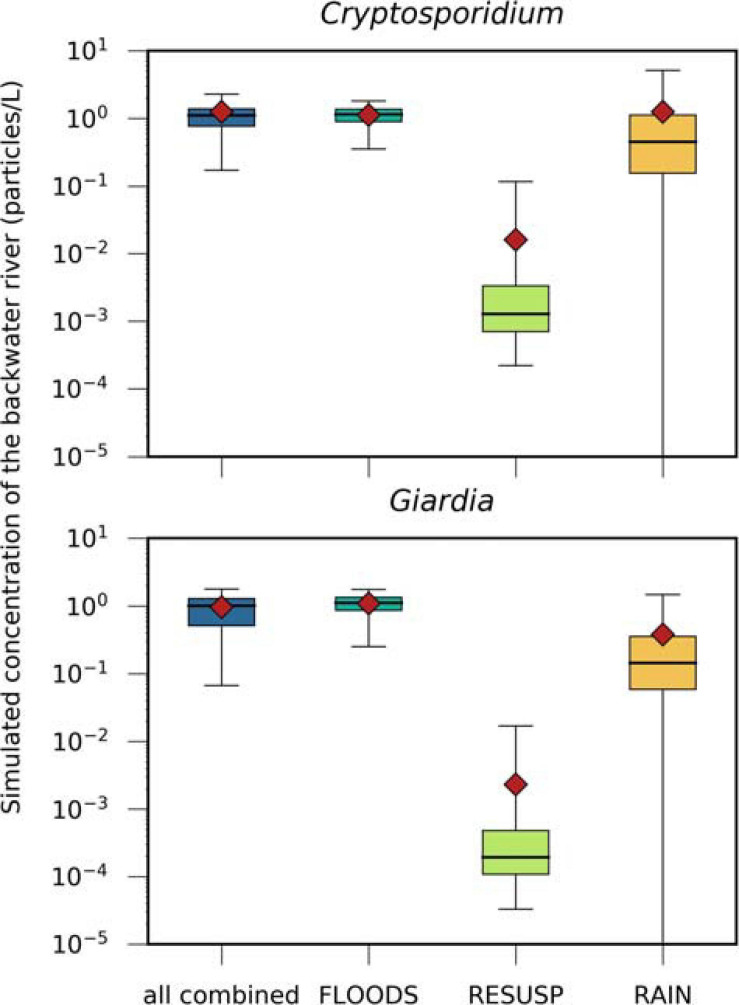
Simulated concentrations of *Cryptosporidium* and *Giardia*, transported via (i) the discharge of Danube water (FLOODS), (ii) the resuspension of pathogens from fecal deposits in inundated areas (RESUSP), (iii) the rainfall-release and runoff from fecal deposits (RAIN), and all scenarios combined. Black horizontal lines, red diamonds, and whiskers mark the median, mean, and 95th percentile values, respectively.

A more detailed definition of how we defined these events is given in the Supplementary Information Section “Detailed Definition of the Events for the Scenarios.” The FLOODS and RAIN scenarios and all scenarios combined resulted in similar ranges of concentrations of *Cryptosporidium* and *Giardia* in the backwater branch (0.4–1.2 particles/L for the mean and 1.8–5.1 particles/L for the 95^*th*^ percentiles, Figure 7). The concentrations were more than one log_10_ lower for the RESUSP scenario. The concentrations were the same for *Giardia* and *Cryptosporidium* for the FLOODS scenario, while they were 70–90 % smaller for *Giardia* than for *Cryptosporidium* for the RAIN and RESUSP scenario due the higher inactivation.

**FIGURE 8:**
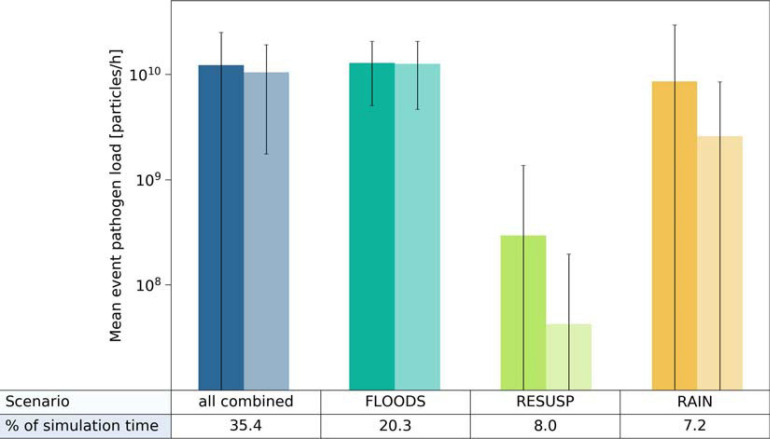
Mean load attribution of *Cryptosporidium* (left columns) and *Giardia* (right columns) via (i) the discharge of Danube water (FLOODS), (ii) the resuspension from fecal deposits in inundated areas (RESUSP), (iii) the rainfall-release and runoff from fecal deposits (RAIN), and all scenarios combined from 2010 to 2015. Whiskers indicate the standard deviations.

We further conducted a source apportionment by calculating the mean pathogen loads of *Cryptosporidium* and *Giardia* in the backwater branch. For that, we multiplied the simulated hourly concentrations by the hourly discharges and evaluated the mean loads for the selected time steps (Section “Scenario Load and Infection Risk Assessment”). The simulated mean loads of *Cryptosporidium* and *Giardia* were again in a similar range for the FLOODS and RAIN scenarios and all scenarios combined (3–13 × 10^9^ particles/h), and were at least one log_10_ lower for the RESUSP scenario. The FLOODS scenario occurred during 20 % of the 6-year time period (Figure 8). The RAIN scenario, which occurred only during 7 % of the time, resulted in higher standard deviations and peaks of loads than the FLOODS scenario in case of *Cryptosporidium*. The RESUSP scenario resulted in the smallest source attribution in comparison, occurring during 8 % of the time.

**FIGURE 9:**
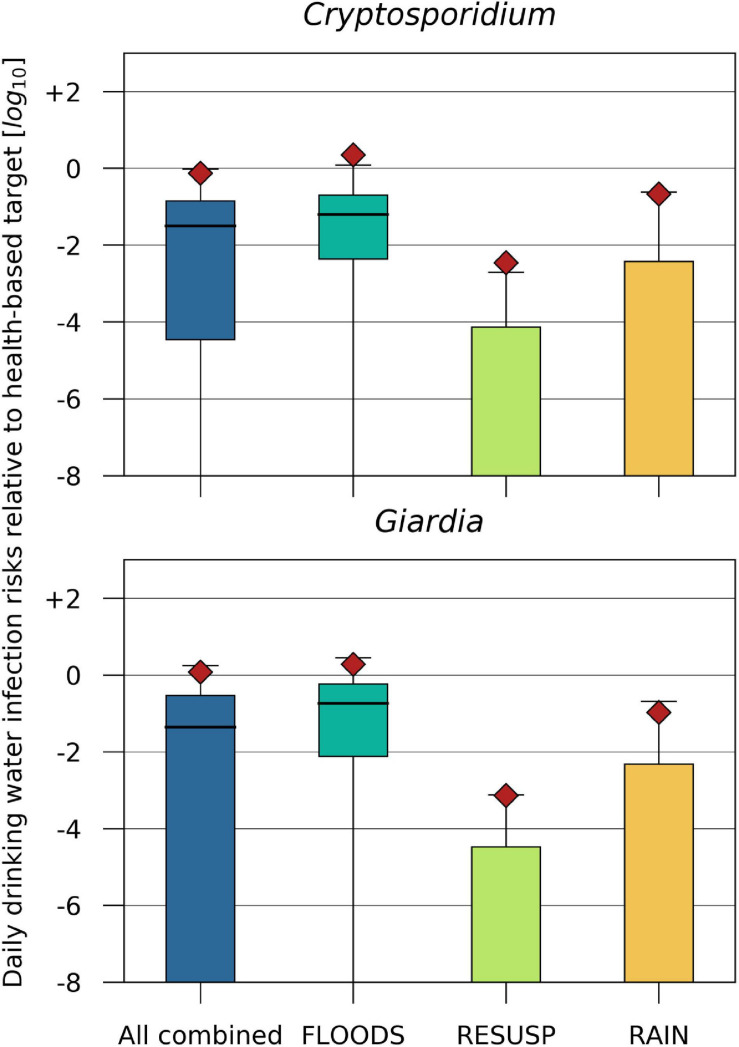
*Cryptosporidium* and *Giardia* daily drinking water infection risks relative to a health-based benchmark of –6 log_10_/person/d (Signor and Ashbolt, 2009) for the scenarios (i) discharge of Danube water (FLOODS), (ii) resuspension from fecal deposits in inundated areas (RESUSP), (iii) rainfall-release and runoff from fecal deposits (RAIN), and all scenarios combined during 2010–2015. The treatment reduction of *Cryptosporidium* and *Giardia* from backwater river water (LRV, Equation 13) was 6.2 and 6.0 log_10_.

The drinking water infection risks relative to a health based target of ≤ 1 ⋅ 10^–6^ infections/person/d were estimated assuming a value of 6.2 and 6.0 as treatment reduction of *Cryptosporidium* and *Giardia* from backwater river water (LRV, Equation 13). The mean drinking water infection risks for *Cryptosporidium* and *Giardia* resulted in values one log_10_ below to close to the health based target for the FLOODS and RAIN scenarios, and all scenarios combined. For the RESUSP scenarios, the mean drinking water infection risks were > 3 log_10_ below the health based target (Figure 9).

## Discussion

### Strengths and Limitations of the Integrative Approach

In this study, we presented a new integrative modeling approach for evaluating the impact of fecal sources and transport pathways on the microbiological quality of a riverine wetland considering safe drinking water. By integrating measured concentrations of human and the most relevant animal MST markers, the approach allowed for the first time quantifying the relative drinking water infection risks from external (allochthonous, i.e., river water inflows) and internal fecal sources (autochthonous, i.e., wild boar, ruminants, birds in the backwater study area). This would not have been possible based on FIO data alone, which are sum indicators in contrast to MST markers (Zhang et al., 2019). The approach also allowed assessing if a given MST marker is appropriate in the study area, given its fecal specificity and fecal sensitivity. These performance characteristics of MST markers can be highly regional- and site-specific (Reischer et al., 2013). Furthermore the required MST performance criteria depend on the relative abundance of the specific fecal sources to be detected amongst the sum of total fecal pollution occurring at the investigation site, e.g., % fraction of human fecal pollution in relation to the sum of human and animal fecal pollution (Reischer et al., 2011). For example, if the animal numbers were different to our study site, the resulting non-target concentrations of the MST markers could render the selected MST marker assays inapplicable. In this case, other markers with appropriate performance characteristics for the specific situation and question have to be chosen. A trade-off between fecal source sensitivity and specificity for MST qPCR assays often exists (Layton et al., 2013; Raith et al., 2013; Mayer et al., 2016). To illustrate this relationship, for recent fecal pollution detection bacterial MST qPCR assays often show high sensitivity but limited specificity as in contrast to many viral qPCR MST assay often showing high source specificity but limited sensitivity (Mayer et al., 2016). As an exemplary test case for the selected human MST marker assay at our study site, we assumed a theoretical increase of the non-target population number of animals by 10 fold, i.e., of ruminants, boar and birds, while leaving the human fecal sources unchanged (Section “Applicability of the MST Marker Specificity for Modeling”). We then simulated the resulting concentration signal of the human MST marker in the backwater river (i.e., the sum of the total qPCR signal from the correct positive (humans) and false negative (animals) DNA targets). The scenarios showed that the selected human MST markers would still be applicable at our study site and for the calibration of the model, even if the animal population number increased drastically by 10-fold (error < 5%, results not shown).

The model was validated in two steps over a 6-year time period, considering (i) individual, presumably important fecal sources using measured concentrations of the respective MST markers, and (ii) all fecal sources combined using measured concentrations of *E. coli*. This was to ascertain that we accounted for the most relevant fecal sources and transport pathways. Interestingly, the measured and simulated MST marker and *E. coli* concentrations were similar, even though other sources of FIO may have potentially contributed (Figure 5). Frick et al. (2018) identified poikilothermic animals (earthworms, gastropods, frogs, and fish) as further potential autochthonous reservoirs of bacterial fecal indicators in our study area. To validate the transient concentration changes during floods and rainfall, it would be advantageous to collect microbial data at high temporal resolution. Current advances in online monitoring techniques may provide this opportunity in the near future (Stadler et al., 2019).

In this study, we modified and extended the microbial fate, transport and infection risk model QMRAcatch (v 1.1 python backwater) to simulate rainfall - runoff and mixing with released microbial particles from animal fecal deposits as functions of transient soil moisture processes according to Bradford and Schijven (2002) and Blöschl et al. (2008). The QMRA framework was fine-tuned for making use of the simulated exposure concentrations of *Cryptosporidium* and *Giardia* at hourly time steps based on human drinking water consumption data (Van Rossum et al., 2011). The discharge rates, volumes and surface water areas simulated by a validated hydrodynamic model allowed accounting for the spatiotemporal changes of these hydrological variables by means of polynomial regression. This was an essential input information for accurately predicting the microbial fate and transport in the alluvial wetland, as pointed out by Sanders et al. (2005) and Liu et al. (2015). Integrating a probabilistic Monte Carlo framework into the model analysis allowed accounting for the uncertainty of the source and transport variables and conducting a microbial infection risk assessment (Liao et al., 2016). One limitation of our model was that it did not account for the microbial particle interaction with the riverbed sediments. While Sanders et al. (2005) showed that the sediment erodibility parameters, and sediment concentrations were important for FIO transport in a coastal wetland, sediment erosion was presumably of minor importance at our study site. Our model predicted 70–80 % of the observed concentrations within acceptable error limits (± 1 log_10_ particles/L), and the simulated and observed cumulative concentrations were not significantly different (Kruskal–Wallis *p* > 0.05). The settling of microbial particles and sediment transport simulations may be included for future applications. To estimate pathogen source loads from animal fecal deposits, we assumed that they were evenly distributed in the backwater area. This simplifying assumption was justifiable in our 14 km^2^ sized model area. In larger wetlands, the spatial distribution of animals may need to be accounted for (Kay et al., 2007).

### Impact of Event-Driven Fecal Pollution Sources and Pathways

The new integrative modeling approach allowed determining the transfer rates of pathogens from diverse fecal sources into wetlands during storm events and floods. Such weather extremes are of increasing concern due to climate change in many parts of the world. Several studies identified links of severe rainfall and flood events to elevated concentrations of pathogens such as *Giardia* and *Cryptosporidium* in rivers (Atherholt et al., 1998), the associated drinking water infection risks (Tolouei et al., 2019), or, to the number of outbreaks and sporadic cases of waterborne illness (Galway et al., 2015; Chhetri et al., 2019). For quantifying the impact of such events on the microbiological water quality of wetlands, modeling frameworks were developed either for pathogen transport via the rainfall-induced release and runoff (Guber et al., 2013), or via floods and resuspension (Sanders et al., 2005; Daniels et al., 2014; Liu et al., 2015). Our integrated modeling approach was developed to quantitatively compare these transfer pathways in a probabilistic framework. Our study showed that rainfall-induced pathogen release from animal fecal deposits, and floods can result in similar ranges of concentrations and loads of *Cryptosporidium* and *Giardia* and required reductions to achieve safe drinking water. This implies for water safety planning, that the autochthonous, homeothermic animal sources, such as ruminants, wild boar and birds, can be similarly important fecal pollution sources as the allochthonous human wastewater. This also implies that additional treatment may be required for drinking water production in wetlands inhabiting abundant wildlife, even in the absence of human wastewater discharges from upstream. According to our estimates, a 5–6 log_10_ reduction of *Cryptosporidium* and *Giardia* is required to achieve safe drinking water during floods and rainfall events. Demeter et al. (2021) considered only human wastewater sources to calculate the required reductions of *Cryptosporidium* to achieve safe drinking water at the Danube study site. Our estimation during floods is 0.5 log_10_ higher due to the additional contribution of diffuse animal sources in the Danube catchment.

For the estimation of infection risks, we used a mixture of beta distributions for the prevalence of the reference pathogens *Cryptosporidium* and *Giardia* in animal waste. We conducted a comprehensive literature survey, and selected values from the most recent, data-intensive studies conducted in temperate, high-income regions as our study area. The human infection risks from the animal fecal sources, however, may still be an overestimate, as we assumed the same dose-response models as for the human wastewater sources. To date, there are no reports about dose-response studies including different genotypes of *Cryptosporidium* or *Giardia* in the scientific literature. However, as long as this information is missing, it seems acceptable for risk assessment to choose this conservative risk assessment approach. Besides the reference pathogens *Cryptosporidium* and *Giardia*, other zoonotic pathogens such as EHEC and *Salmonella* spp. could be included in future analysis. These bacteria are also important reference pathogens occurring both in human and animal sources (Stalder et al., 2011), and their effects will depend on region and microorganism-specific source concentrations, prevalence and decay.

The modeling approach is transferrable to other riverine wetlands worldwide, even though the results of our study are site-specific. To support water safety planning, it is important to integrate site-specific data into the modeling analysis and to validate the different transfer pathways of pathogens. In contrast to our local-scale approach, larger scale modeling studies previously identified hot spots of fecal pollution or evaluated the impact of system changes on the microbiological water quality (Medema and Schijven, 2001; Vermeulen et al., 2015; Sterk et al., 2016). These studies commonly made generalizing assumptions about the pathogen source and transport parameters as well as the hydrological and environmental boundary conditions and were not validated on real-world data.

## Conclusion

• This study presents a new integrative modeling approach for determining the transfer rates of pathogens from diverse fecal sources into alluvial wetlands during storm events and floods considering safe drinking water supply.
• The modified and extended QMRAcatch (v1.1 Python backwater) combines microbial source tracking (MST) with 2-D hydrodynamic flow, rainfall-runoff, microbial fate and transport, and QMRA.
• The modeling approach allowed assessing the applicability of the chosen MST markers for the targeted fecal pollution source in relation to the total sum of all fecal pollution sources, considering fecal sensitivity and fecal specificity. They were found fully applicable for the modeling requirements and the research question in this study. The model captured the most relevant fecal sources and transport pathways, as proven by the model validation based on MST markers and *E. coli*.
• Allochthonous and autochthonous fecal sources during floods and rainfall events contributed similar ranges of concentrations and loads of *Cryptosporidium* and *Giardia* in the backwater branch, and drinking water infection risks relative to a health-based target.

## Data Availability Statement

The original contributions presented in the study are included in the article/Supplementary Material, further inquiries can be directed to the corresponding author/s.

## Author Contributions

JD extended the computer code of QMRAcatch, did the computational and model analyses, and wrote the manuscript. KD contributed to the design of the study. KD and RL organized the microbial source tracking database. RL, SC-A, and KD did the molecular and microbiological analyses, supported by RS and JW. GL took the water samples in the field. GS conducted the literature survey on pathogen prevalence and source concentrations. JS gave support with QMRA modeling and provided data on the cumulative doses to calculate daily drinking water infection risks. JK did the hydrodynamic model simulations and provided support regarding the hydrological model. SC-A, RS, JW, AK, AB, and AF contributed to conception and in the acquisition of funding support. SC-A and KD wrote sections of the manuscript. All authors contributed to manuscript revision, read, and approved the submitted version.

## Conflict of Interest

The authors declare that the research was conducted in the absence of any commercial or financial relationships that could be construed as a potential conflict of interest.

## References

[B1] Ahmed W. Zhang Q. Kozak S. Beale D. Gyawali P. Sadowsky M. J. (2019). Comparative decay of sewage-associated marker genes in beach water and sediment in a subtropical region. *Water Res.* 149 511–521. 10.1016/j.watres.2018.10.088 30500686

[B2] Arnberger A. Frey-Roos F. Eder R. Muralt G. Nopp-Mayr U. Tomek H. (2009). *Ökologische und soziale Tragfähigkeiten als Managementherausforderungen für suburbane Biosphärenparke am Beispiel Untere Lobau (Ecological and Social Carrying Capacities as Management Challenges for Peri-Urban Biosphere Reserves). Final report.* Vienna: University of Natural Resources and Life Sciences.

[B3] Atherholt T. B. LeChevallier M. W. Norton W. D. Rosen J. S. (1998). Effect of rainfall on Giardia and Crypto. *J. Am. Water Works Assoc.* 90 66–80. 10.1002/j.1551-8833.1998.tb08499.x

[B4] Bertrand I. Schijven J. F. Sanchez G. Wyn-Jones P. Ottoson J. Morin T. (2012). The impact of temperature on the inactivation of enteric viruses in food and water: a review. *J. Appl. Microbiol.* 112 1059–1074. 10.1111/j.1365-2672.2012.05267.x 22380614

[B5] Blöschl G. Reszler C. Komma J. (2008). A spatially distributed flash flood forecasting model. *Environ. Mod. Softw.* 23 464–478. 10.1016/j.envsoft.2007.06.010

[B6] Böhm J. (2016). *Grundlagen für die Optimierung des Wildmanagements im Nationalpark Donau-AuenEin Vergleich verschiedener Schutzgebiete unter besonderer Berücksichtigung des Managements von Wildruhegebieten.* San Francisco, CA: Wildtierökologie und Wildtiermanagement, University of Natural Applied Sciences (BOKU).

[B7] Boyer D. G. Kuczynska E. Fayer R. (2009). Transport, fate, and infectivity of *Cryptosporidium parvum* oocysts released from manure and leached through macroporous soil. *Environ. Geol.* 58 1011–1019. 10.1007/s00254-008-1580-x

[B8] Bradford S. A. Schijven J. (2002). Release of *Cryptosporidium* and *Giardia* from dairy calf manure: impact of solution salinity. *Environ. Sci. Technol.* 36 3916–3923. 10.1021/es025573l 12269743

[B9] Castro-Hermida J. A. Garcia-Presedo I. Gonzalez-Warleta M. Mezo M. (2011). Prevalence of *Cryptosporidium* and *Giardia* in roe deer (*Capreolus capreolus*) and wild boars (*Sus scrofa*) in Galicia (NW, Spain). *Veterinary Parasitol.* 179 216–219. 10.1016/j.vetpar.2011.02.023 21429669

[B10] Chhetri B. K. Galanis E. Sobie S. Brubacher J. Balshaw R. Otterstatter M. (2019). Projected local rain events due to climate change and the impacts on waterborne diseases in Vancouver, British Columbia, Canada. *Environ. Health* 18:116. 10.1186/s12940-019-0550-y 31888648 PMC6937929

[B11] Daniels M. E. Hogan J. Smith W. A. Oates S. C. Miller M. A. Hardin D. (2014). Estimating environmental conditions affecting protozoal pathogen removal in surface water wetland systems using a multi-scale, model-based approach. *Sci. Total Environ.* 493 1036–1046. 10.1016/j.scitotenv.2014.06.053 25016109

[B12] de Regnier D. P. Cole L. Schupp D. G. Erlandsen S. L. (1989). Viability of *Giardia* cysts suspended in lake, river, and tap water. *Appl. Environ. Microbiol.* 55 1223–1229. 10.1128/aem.55.5.1223-1229.1989 2757381 PMC184281

[B13] Demeter K. Derx J. Komma J. Parajka J. Schijven J. Sommer R. (2021). Modelling the interplay of future changes and wastewater management measures on the microbiological river water quality considering safe drinking water production. *Sci. Total Environ.* 768:144278. 10.1016/j.scitotenv.2020.144278 33736313

[B14] Derx J. Schijven J. Sommer R. Zoufal-Hruza C. M. van Driezum I. H. Reischer G. (2016). QMRAcatch: human-associated fecal pollution and infection risk modeling for a river/floodplain environment. *J. Environm. Q.* 45 1205–1214. 10.2134/jeq2015.11.0560 27380068

[B15] Dorner S. M. Huck P. M. Slawson R. M. (2004). Estimating potential environmental loadings of *Cryptosporidium* spp. and *Campylobacter* spp. from livestock in the Grand River Watershed, Ontario, Canada. *Environ. Sci. Technol.* 38 3370–3380. 10.1021/es035208 15260337

[B16] European Commission (2018). *Urban Waste Water Website: Austria.* Available online at: http://uwwtd.oieau.fr/Austria/ (accessed May 25, 2018).

[B17] Farnleitner A. Derx J. Frick C. Reiner P. Savio D. Zoufal-Hruza C. (2014). *Water Connection (New) Danube. Lower Lobau (Nationalpark Donauauen), Water Quality Report for Microbiology/Water Hygiene.* Vienna: Municipal Department MA45.

[B18] Farnleitner A. H. Ryzinska-Paier G. Reischer G. H. Burtscher M. M. Knetsch S. Kirschner A. K. T. (2010). *Escherichia coli* and enterococci are sensitive and reliable indicators for human, livestock and wildlife faecal pollution in alpine mountainous water resources. *J. Appl. Microbiol.* 109 1599–1608. 10.1111/j.1365-2672.2010.04788.x 20629798 PMC3154642

[B19] Franz E. Schijven J. Husman A. M. D. Blaak H. (2014). Meta-regression analysis of commensal and pathogenic *Escherichia coli* survival in soil and water. *Environ. Sci. Technol.* 48 6763–6771. 10.1021/es501677c 24839874

[B20] Frick C. Vierheilig J. Linke R. Savio D. Zornig H. Antensteiner R. (2018). Poikilothermic animals as a previously unrecognized source of fecal indicator bacteria in a backwater ecosystem of a large river. *Appl. Environ. Microbiol.* 84:aem.00715-00718. 10.1128/aem.00715-18 29884761 PMC6070746

[B21] Frick C. Vierheilig J. Nadiotis-Tsaka T. Ixenmaier S. Linke R. Reischer G. H. (2020). Elucidating fecal pollution patterns in alluvial water resources by linking standard fecal indicator bacteria to river connectivity and genetic microbial source tracking. *Water Res.* 184:116132. 10.1016/j.watres.2020.116132 32777635

[B22] Frühauf J. Sabathy E. (2006a). Untersuchung an Schilf- und Wasservögeln in der unteren Lobau, Teil I: Bestände und Habitat. *Wissenschaftliche Reihe* 23 1–68. 10.1515/9783111624150-002

[B23] Frühauf J. Sabathy E. (2006b). Untersuchung an Schilf- und Wasservögeln in der unteren Lobau, Teil II: Arten. *Wissenschaftliche Reihe* 24 1–80. 10.1515/9783486784503-004

[B24] Gabriel H. Blaschke A. P. Taschke R. Mayr E. (2014). *Water Connection (New) DanubeLower Lobau (Nationalpark Donauauen), Water Quantity Report for Surface Water.* Vienna: Municipial Department MA45 Vienna Waters.

[B25] Galway L. P. Allen D. M. Parkes M. W. Li L. Takaro T. K. (2015). Hydroclimatic variables and acute gastro-intestinal illness in British Columbia, Canada: a time series analysis. *Water Resour. Res.* 51 885–895. 10.1002/2014WR015519

[B26] Garcia-Presedo I. Pedraza-Diaz S. Gonzalez-Warleta M. Mezo M. Gomez-Bautista M. Ortega-Mora L. M. (2013). The first report of *Cryptosporidium bovis*, *C. ryanae* and *Giardia duodenalis* sub-assemblage A-II in roe deer (*Capreolus capreolus*) in Spain. *Veter. Parasitol.* 197 658–664. 10.1016/j.vetpar.2013.07.002 23890824

[B27] Graczyk T. K. Fayer R. Trout J. M. Lewis E. J. Farley C. A. Sulaiman I. (1998). *Giardia* sp. cysts and infectious *Cryptosporidium parvum* oocysts in the feces of migratory Canada geese (*Branta canadensis*). *Appl. Environ. Microbiol.* 64 2736–2738. 10.1128/aem.64.7.2736-2738.1998 9647860 PMC106456

[B28] Grant S. B. Sanders B. F. Boehm A. B. Redman J. A. Kim J. H. Mrše R. D. (2001). Generation of enterococci bacteria in a coastal saltwater marsh and its impact on surf zone water quality. *Environ. Sci. Technol.* 35 2407–2416. 10.1021/es0018163 11432541

[B29] Green H. C. Haugland R. A. Varma M. Millen H. T. Borchardt M. A. Field K. G. (2014). Improved HF183 quantitative real-time PCR assay for characterization of human fecal pollution in ambient surface water samples. *Appl. Environ. Microbiol.* 80 3086–3094. 10.1128/AEM.04137-13 24610857 PMC4018914

[B30] Guber A. K. Fry J. Ives R. L. Rose J. B. (2015). *Escherichia coli* survival in, and release from, white-tailed deer feces. *Appl. Environ. Microbiol.* 81 1168–1176. 10.1128/Aem.03295-14 25480751 PMC4292497

[B31] Guber A. K. Pachepsky Y. A. Dao T. H. Shelton D. R. Sadeghi A. M. (2013). Evaluating manure release parameters for nonpoint contaminant transport model KINEROS2/STWIR. *Ecol. Mod.* 263 126–138. 10.1016/j.ecolmodel.2013.05.008

[B32] Hahn S. Bauer S. Klaassen M. (2007). Estimating the contribution of carnivorous waterbirds to nutrient loading in freshwater habitats. *Fresh. Biol.* 52 2421–2433. 10.1111/j.1365-2427.2007.01838.x

[B33] Hein T. Blaschke A. P. Haidvogl G. Hohensinner S. Kucera-Hirzinger V. Preiner S. (2006). Optimised management strategies for the Biosphere reserve Lobau, Austria, based on a multi criteria decision support system. *Ecohydrol. Hydrobiol.* 6 25–36. 10.1016/S1642-3593(06)70123-9

[B34] Helsel D. R. (2006). Fabricating data: how substituting values for nondetects can ruin results, and what can be done about it. *Chemosphere* 65 2434–2439. 10.1016/j.chemosphere.2006.04.051 16737727

[B35] Hinterberger B. Arnberger A. Brandenburg C. Cermak P. (2000). *Besucherstromanalyse für den Wiener Bereich des Nationalpark Donau-Auen - Lobau: GIS-Implementierung und erste Ergebnisse.* Wien: Magistrat der Stadt Wien, Magistratsabteilung, 49.

[B36] Hogan J. N. Daniels M. E. Watson F. G. Conrad P. A. Oates S. C. Miller M. A. (2012). Longitudinal poisson regression to evaluate the epidemiology of *Cryptosporidium, Giardia*, and fecal indicator bacteria in coastal California wetlands. *Appl. Environ. Microbiol.* 78 3606–3613. 10.1128/AEM.00578-12 22427504 PMC3346375

[B37] Hong E. M. Park Y. Muirhead R. Jeong J. Pachepsky Y. A. (2018). Development and evaluation of the bacterial fate and transport module for the Agricultural Policy/Environmental eXtender (APEX) model. *Sci. Total Environ.* 615 47–58. 10.1016/j.scitotenv.2017.09.231 28963896

[B38] ISO (2006). ISO 1553:Water Quality Isolation and Identification of *Cryptosporidium* oocysts and *Giardia* cysts from water. Technical Committee: ISO/TC 147/SC 4 Microbiological methods, ICS : 07.100.20 Microbiology of water.

[B39] ISO (2001). ISO 16649-1: Microbiology of food and animal feeding stuffs – Horizontal method for the enumeration of beta-glucuronidase-positive Escherichia coli – Part 1: Colony-count technique at 44 degrees C using membranes and 5-bromo-4-chloro-3-indolyl beta-D-glucuronide. Technical Committee : ISO/TC 34/SC 9 Microbiology, ICS : 07.100.30 Food microbiology.

[B40] Ives R. L. Kamarainen A. M. John D. E. Rose J. B. (2007). Use of cell culture to assess *Cryptosporidium parvum* survival rates in natural groundwaters and surface waters. *Appl. Environ. Microbiol.* 73 5968–5970. 10.1128/Aem.00347-07 17675439 PMC2074924

[B41] Kay D. Aitken M. Crowther J. Dickson I. Edwards A. C. Francis C. (2007). Reducing fluxes of faecal indicator compliance parameters to bathing waters from diffuse agricultural sources: the Brighouse Bay study, Scotland. *Environ. Pollut.* 147 138–149. 10.1016/j.envpol.2006.08.019 17055631

[B42] Kirschner A. K. T. Reischer G. H. Jakwerth S. Savio D. Ixenmaier S. Toth E. (2017). Multiparametric monitoring of microbial faecal pollution reveals the dominance of human contamination along the whole Danube river. *Water Res.* 124 543–555. 10.1016/j.watres.2017.07.052 28806705 PMC5718294

[B43] Kobayashi A. Sano D. Hatori J. Ishii S. Okabe S. (2013). Chicken and duck-associated *Bacteroides*-Prevotella genetic markers for detecting fecal contamination in environmental water. *Appl. Microbiol. Biotechnol.* 97 7427–7437. 10.1007/s00253-012-4469-2 23053113

[B44] Layton B. A. Cao Y. Ebentier D. L. Hanley K. Ballesté E. Brandão J. (2013). Performance of human fecal anaerobe-associated PCR-based assays in a multi-laboratory method evaluation study. *Water Res.* 47 6897–6908. 10.1016/j.watres.2013.05.060 23992621

[B45] Liao H. Krometis L.-A. H. Kline K. (2016). Coupling a continuous watershed-scale microbial fate and transport model with a stochastic dose-response model to estimate risk of illness in an urban watershed. *Sci. Total Environ.* 55 668–675. 10.1016/j.scitotenv.2016.02.044 26897410

[B46] Liu W.-C. Chan W.-T. Young C.-C. (2015). Modeling fecal coliform contamination in a tidal Danshuei river estuarine system. *Sci. Total Environ.* 502 632–640. 10.1016/j.scitotenv.2014.09.065 25302451

[B47] Mayer R. E. Sofill-Mas S. Egle L. Reischer G. H. Schade M. Fernandez-Cassi X. (2016). Occurrence of human-associated *Bacteroidetes genetic* source tracking markers in raw and treated wastewater of municipal and domestic origin and comparison to standard and alternative indicators of faecal pollution. *Water Res.* 90 265–276. 10.1016/j.watres.2015.12.031 26745175 PMC4884448

[B48] Medema G. J. Schijven J. F. (2001). Modelling the sewage discharge and dispersion of *Cryptosporidium* and *Giardia* in surface water. *Water Res.* 35 4307–4316. 10.1016/S0043-1354(01)00161-0 11763032

[B49] Mieszkin S. Furet J. P. Corthier G. Gourmelon M. (2009). Estimation of pig fecal contamination in a river catchment by real-time PCR using two pig-specific Bacteroidales 16S rRNA genetic markers. *Appl. Environ. Microbiol.* 75 3045–3054. 10.1128/AEM.02343-08 19329663 PMC2681621

[B50] Nash J. E. Sutcliffe J. V. (1970). River flow forecasting through conceptual models part Ia discussion of principles. *J. Hydrol.* 10 282–290. 10.1016/0022-1694(70)90255-6

[B51] Oladeinde A. Bohrmann T. Wong K. Purucker S. T. Bradshaw K. Brown R. (2014). Decay of fecal indicator bacterial populations and bovine-associated source-tracking markers in freshly deposited cow pats. *Appl. Environ. Microbiol.* 80 110–118. 10.1128/aem.02203-13 24141130 PMC3910999

[B52] Olson M. E. Goh J. Phillips M. Guselle N. McAllister T. A. (1999). *Giardia* cyst and *Cryptosporidium* oocyst survival in water, soil, and cattle feces. *J. Environ. Q.* 28 1991–1996. 10.2134/jeq1999.00472425002800060040x

[B53] Parz-Gollner R. (2006). Zur Situation der Kormoranschlafplätze im Nationalpark Donau-Auen (NÖ) - Auswirkungen der Uferrückbauten im Bereich des Schlafplatzes Turnhaufen. Universität für Bodenkultur, Department für Integrative Biologie und Biodiversitätsforschung>, Vienna, Austria.

[B54] Pavlik M. Wildeman T. Kohn K. Emerick J. Robinson R. (1999). Fate and transport of metals in a natural wetland receiving mine drainage. *J. Am. Soc. Mining Reclamat.* 1999 563–578. 10.21000/JASMR99010563

[B55] Peterson E. W. Hanna L. A. (2016). Estrogen reduction in a coupled wetland and ground water flow-through system. *Environ. Earth Sci.* 75:384. 10.1007/s12665-016-5259-4

[B56] Raith M. R. Kelty C. A. Griffith J. F. Schriewer A. Wuertz S. Mieszkin S. (2013). Comparison of PCR and quantitative real-time PCR methods for the characterization of ruminant and cattle fecal pollution sources. *Water Res.* 47 6921–6928. 10.1016/j.watres.2013.03.061 23871256

[B57] Reckendorfer W. Funk A. Gschöpf C. Hein T. Schiemer F. (2013). Aquatic ecosystem functions of an isolated floodplain and their implications for flood retention and management. *J. Appl. Ecol.* 50 119–128. 10.1111/1365-2664.12029

[B58] Regli S. Rose J. B. Haas C. N. Gerba C. P. (1991). Modeling the risk from giardia and viruses in drinking-water. *J. Am. Water Works Assoc.* 83 76–84. 10.1002/j.1551-8833.1991.tb07252.x

[B59] Reischer G. H. Ebdon J. E. Bauer J. M. Schuster N. Ahmed W. Astrom J. (2013). Performance characteristics of qPCR assays targeting human- and ruminant-associated bacteroidetes for microbial source tracking across sixteen countries on six continents. *Environ. Sci. Technol.* 47 8548–8556. 10.1021/es304367t 23755882 PMC3737603

[B60] Reischer G. H. Haider J. M. Sommer R. Stadler H. Keiblinger K. M. Hornek R. (2008). Quantitative microbial faecal source tracking with sampling guided by hydrological catchment dynamics. *Environ. Microbiol.* 10 2598–2608. 10.1111/j.1462-2920.2008.01682.x 18564182 PMC3025520

[B61] Reischer G. H. Kasper D. C. Steinborn R. Farnleitner A. H. Mach R. L. (2007). A quantitative real-time PCR assay for the highly sensitive and specific detection of human faecal influence in spring water from a large alpine catchment area. *Lett. Appl. Microbiol.* 44 351–356. 10.1111/j.1472-765X.2006.02094.x 17397471 PMC3068607

[B62] Reischer G. H. Kasper D. C. Steinborn R. Mach R. L. Farnleitner A. H. (2006). Quantitative PCR method for sensitive detection of ruminant fecal pollution in freshwater and evaluation of this method in alpine karstic regions. *Appl. Environ. Microbiol.* 72 5610–5614. 10.1128/Aem.00364-06 16885315 PMC1538736

[B63] Reischer G. H. Kollanur D. Vierheilig J. Wehrspaun C. Mach R. L. Sommer R. (2011). Hypothesis-driven approach for the identification of fecal pollution sources in water resources. *Environ. Sci. Technol.* 45 4038–4045. 10.1021/es103659s 21466151 PMC3084580

[B64] Sanders B. F. Arega F. Sutula M. (2005). Modeling the dry-weather tidal cycling of fecal indicator bacteria in surface waters of an intertidal wetland. *Water Res.* 39 3394–3408. 10.1016/j.watres.2005.06.004 16051310

[B65] Schijven J. Derx J. Husman A. M. D. Blaschke A. P. Farnleitner A. H. (2015). QMRAcatch: microbial quality simulation of water resources including infection risk assessment. *J. Environ. Q.* 44 1491–1502. 10.2134/jeq2015.01.0048 26436266 PMC4884445

[B66] Schijven J. F. Teunis P. P. M. Rutjes S. A. Bouwknegt M. Husman A. M. D. (2011). QMRAspot: a tool for quantitative microbial risk assessment from surface water to potable water. *Water Res.* 45 5564–5576. 10.1016/j.watres.2011.08.024 21885080

[B67] Schmidt M. Sommer K. Kriebitzsch W. U. Ellenberg H. von Oheimb G. (2004). Dispersal of vascular plants by game in northern Germany. part I: roe deer (*Capreolus capreolus*) and wild boar (*Sus scrofa*). *Eur. J. Forest Res.* 123 167–176. 10.1007/s10342-004-0029-3

[B68] Schreiber H. Behrendt H. Constantinescu L. T. Cvitanic I. Drumea D. Jabucar D. (2005). Nutrient emissions from diffuse and point sources into the River Danube and its main tributaries for the period of 1998–2000 results and problems. *Water Sci. Technol.* 51 283–290. 10.2166/wst.2005.0602 15850201

[B69] Schulze C. H. Schütz C. (2013). Gewässervernetzung (Neue) Donau Untere Lobau (Nationalpark Donau Auen). Wissenschaftliche Beweissicherung Lausgrund: Erhebung der Brutvögel mit Gewässerbindung 2012 (Unpublished Project Report). Wasser Cluster Lunz.

[B70] Signor R. S. Ashbolt N. J. (2009). Comparing probabilistic microbial risk assessments for drinking water against daily rather than annualised infection probability targets. *J. Water Health* 7 535–543. 10.2166/wh.2009.101 19590121

[B71] Stadler P. Loken L. C. Crawford J. T. Schramm P. J. Sorsa K. Kuhn C. (2019). Spatial patterns of enzymatic activity in large water bodies: Ship-borne measurements of beta-D-glucuronidase activity as a rapid indicator of microbial water quality. *Sci. Total Environ.* 651 1742–1752. 10.1016/j.scitotenv.2018.10.084 30316092

[B72] Stalder G. L. Farnleitner A. Sommer R. Beiglbock C. Walzer C. (2011). Hazard- and risk based concepts for the assessment of microbiological water quality-part 2. *Wiener Tierarztliche Monatsschrift* 98 54–65.

[B73] Sterk A. Schijven J. Husman A. M. D. de Nijs T. (2016). Effect of climate change on runoff of *Campylobacter* and *Cryptosporidium* from land to surface water. *Water Res.* 95 90–102. 10.1016/j.watres.2016.03.005 26986498

[B74] Stevenson M. E. Blaschke A. P. Toze S. Sidhu J. P. Ahmed W. van Driezum I. H. (2015). Biotin- and glycoprotein-coated microspheres as surrogates for studying filtration removal of *Cryptosporidium parvum* in a granular limestone aquifer medium. *Appl. Environ. Microbiol.* 81 4277–4283. 10.1128/AEM.00885-15 25888174 PMC4475872

[B75] Teunis P. F. Havelaar A. H. (2000). The beta poisson dose-response model is not a single-hit model. *Risk Anal.* 20 513–520. 10.1111/0272-4332.204048 11051074

[B76] Tolouei S. Dewey R. Snodgrass W. J. Edge T. A. Andrews R. C. Taghipour M. (2019). Assessing microbial risk through event-based pathogen loading and hydrodynamic modelling. *Sci. Total Environ.* 693:133567. 10.1016/j.scitotenv.2019.07.373 31374504

[B77] van Elsas J. D. Semenov A. V. Costa R. Trevors J. T. (2011). Survival of *Escherichia coli* in the environment: fundamental and public health aspects. *ISME J.* 5 173–183. 10.1038/ismej.2010.80 20574458 PMC3105702

[B78] Van Rossum C. T. M. Fransen H. P. Verkaik-Kloosterman J. Buurma-Rethans E. J. M. Ocké M. C. (2011). Durch National Food Consumption Survey 2007-2010: Diet of Children and Adults Aged 7 to 69 Years. *National Institute of Public Health and the Environment (RIVM) Report 350050006/2011.*

[B79] Vermeulen L. C. de Kraker J. Hofstra N. Kroeze C. Medema G. (2015). Modelling the impact of sanitation, population growth and urbanization on human emissions of *Cryptosporidium* to surface waters-a case study for Bangladesh and India. *Environ. Res. Lett.* 10:094017. 10.1088/1748-9326/10/9/094017

[B80] Vierheilig J. Frick C. Mayer R. E. Kirschner A. K. T. Reischer G. H. Derx J. (2013). *Clostridium perfringens* is not suitable for the indication of fecal pollution from ruminant wildlife but is associated with excreta from nonherbivorous animals and human sewage. *Appl. Environ. Microbiol.* 79 5089–5092. 10.1128/Aem.01396-13 23747707 PMC3754692

[B81] von Oheimb G. Schmidt M. Kriebitzsch W.-U. Ellenberg H. (2005). Dispersal of vascular plants by game in northern Germany. part II: red deer (*Cervus elaphus*). *Eur. J. Forest Res.* 124 55–65. 10.1007/s10342-005-0053-y

[B82] Willmott C. J. Matsuura K. (2005). Advantages of the mean absolute error (MAE) over the root mean square error (RMSE) in assessing average model performance. *Clim. Res.* 30 79–82. 10.3354/cr030079

[B83] Wu B. Wang C. Zhang C. Sadowsky M. J. Dzakpasu M. Wang X. C. (2020). Source-associated gastroenteritis risk from swimming exposure to aging fecal pathogens. *Environ. Sci. Technol.* 54 921–929. 10.1021/acs.est.9b01188 31800232

[B84] Zhang Q. Gallard J. Wu B. Harwood V. J. Sadowsky M. J. Hamilton K. A. (2019). Synergy between quantitative microbial source tracking (qMST) and quantitative microbial risk assessment (QMRA): a review and prospectus. *Environ. Internat.* 130:104703. 10.1016/j.envint.2019.03.051 31295713

