# Corrigendum: Genetic microbial source tracking support QMRA modeling for a riverine wetland drinking water resource

**DOI:** 10.3389/fmicb.2022.973379

**Published:** 2022-07-18

**Authors:** Julia Derx, Katalin Demeter, Rita Linke, Sílvia Cervero-Aragó, Gerhard Lindner, Gabrielle Stalder, Jack Schijven, Regina Sommer, Julia Walochnik, Alexander K. T. Kirschner, Jürgen Komma, Alfred P. Blaschke, Andreas H. Farnleitner

**Affiliations:** ^1^Institute of Hydraulic Engineering and Water Resources Management, TU Wien, Vienna, Austria; ^2^Research Group Environmental Microbiology and Molecular Diagnostics E166/5/3, Institute of Chemical, Environmental and Bioscience Engineering, TU Wien, Vienna, Austria; ^3^Institute for Hygiene and Applied Immunology, Medical University of Vienna, Vienna, Austria; ^4^Institute of Wildlife Ecology, University of Veterinary Medicine, Vienna, Austria; ^5^Department of Statistics, Informatics and Modelling, National Institute for Public Health and the Environment (RIVM), Bilthoven, Netherlands; ^6^Faculty of Geosciences, Department of Earth Sciences, Utrecht University, Utrecht, Netherlands; ^7^Institute of Specific Prophylaxis and Tropical Medicine, Medical University of Vienna, Vienna, Austria; ^8^Division Water Quality and Health, Department of Pharmacology, Physiology, and Microbiology, Karl Landsteiner University of Health Sciences, Krems an der Donau, Austria

**Keywords:** genetic microbial source tracking markers, microbial fate and transport model, hydrodynamic model, *Cryptosporidium*, *Giardia*, QMRA, microbial decay in environment

In the published article, there was an error in affiliation 1.

Instead of “Institute of Hydraulic Engineering and Water Resources Management, Vienna, Austria,” it should be “Institute of Hydraulic Engineering and Water Resources Management, TU Wien, Vienna, Austria.”

In the published article, there was an error in affiliation 2.

Instead of “Research Group Environmental Microbiology and Molecular Diagnostics E166/5/3, Institute of Chemical, Environmental and Bioscience Engineering, Vienna, Austria,” it should be “Research Group Environmental Microbiology and Molecular Diagnostics E166/5/3, Institute of Chemical, Environmental and Bioscience Engineering, TU Wien, Vienna, Austria.”

The authors apologize for these errors and state that this does not change the scientific conclusions of the article in any way. The original article has been updated.

## Publisher's note

All claims expressed in this article are solely those of the authors and do not necessarily represent those of their affiliated organizations, or those of the publisher, the editors and the reviewers. Any product that may be evaluated in this article, or claim that may be made by its manufacturer, is not guaranteed or endorsed by the publisher.

