# Supplementary material for: Genetic Microbial Source Tracking Support QMRA Modeling for a Riverine Wetland Drinking Water Resource
Source: Front Microbiol. 2021 Jul 14;12:668778. doi: 10.3389/fmicb.2021.668778 (PMC8317494; doi:10.3389/fmicb.2021.668778)
Supplement: Supplementary file 1 [file Data_Sheet_1.docx]

Supplementary Material

# Supplementary Data

## Prevalence and concentration of microorganisms in faeces

Supplementary Table 1 shows the statistics of the literature data that were used to determine the mixture of beta distributions for prevalence data of both *Cryptosporidium* and *Giardia* (see also (Dorner et al., 2004) and (Sterk et al., 2016). Parameters α and β in equation (4) are determined using:

α = s + 1 (1)

β = n - s + 1 (2)

Where s is the number of positive animals and n is the number of animal samples.

## Detailed model information

The transient water quantities were determined by means of polynomial regression based on the hydrodynamic flow simulations during the rising limb of a 9-yearly flood event in January 2011 (Supplementary Table 2). For determining the discharge of river water into the backwater river (Q_bw_), we compared the observed Danube discharges (Q_r_) during the rising limb of the flood event in January 2011 with the simulated discharges at the entry point of the Danube into the backwater river (observation point LSW2, Figure 2). The discharge volumes (V_bw_) and inundated areas and volumes (A_fl_, V_fl_) were determined by spatial mesh integration of the simulated water depths and surface water elevations using a triangular mesh interpolation method (Surfer 12, Golden Software). The regression equations were determined by comparing the observed Danube discharge with the calculated discharge volumes and inundated areas and volumes during the rising limb of the flood event. The polynomial regression equations were then used to determine the discharges and volumes of the backwater river, and the volumes and areas of inundation over the simulation time during 2010 – 2015, if the Danube exceeded a discharge of 2200 m^3^/s. At discharges lower than that, the discharge and volume of the backwater river were set to 0.0 m^3^/s and 429600 m^3^, respectively, and the inundated area and inundated volume were set to 645800 m^2^ and 440800 m^3^, respectively.

## Detailed definition of the events for the scenarios

This section gives a more detailed information of how we defined the events for evaluating the scenarios (Section 3.3). The FLOODS scenario was defined for the case that the discharge of the backwater (Q_BW_) was greater than 0 m^3^/s. The RESUSP scenario was defined for the case when the flooded area increased from one time step to the next one by a certain threshold (1-$\Delta$A_dep_ > 1 $\cdot$ 10^-6^, Equation 6). The RAIN scenario was defined for days when the rainfall was greater than 0 mm.

# Supplementary Tables

Table 1: Prevalence data for *Cryptosporidium* spp. and *Giardia* (oo)cysts for the different sources; s is the number of infected animals and n is the number of animal samples.

|  | **Prevalence *Cryptosporidium*** | | | | **Prevalence *Giardia*** | | | |
| --- | --- | --- | --- | --- | --- | --- | --- | --- |
| **Input source** | **s** | **n** | **Species** | **Ref** | **s** | **n** | **Species** | **Ref** |
| Dog | 8 | 64 |  | (Bajer and Bednarska 2007 | 15 | 1001 |  | (Hinney et al., 2017) |
|  | 8 | 152 |  | (Overgaauw et al., 2009) | 11 | 325 |  | (Hinney et al., 2017) |
|  |  |  |  |  | 22 | 151 |  | (Nikolic et al., 2008) |
|  |  |  |  |  | 18 | 350 |  | (Zygner et al., 2006) |
|  |  |  |  |  | 14 | 161 |  | (Overgaauw et al., 2009) |
| Deer | 14 | 341 |  | (Feng, 2010) | 2 | 118 |  | (Paziewska et al., 2007) |
|  | 2 | 196 |  | (Trogu, 2015) | 6 | 196 | *G.duodenalis* ass. A | (Trogu, 2015) |
|  | 21 | 416 |  | (Trogu, 2015) | 10 | 119 | *G. duodenalis* ass. A | (Trogu, 2015) |
|  | 4 | 119 |  | (Trogu, 2015) | 8 | 212 | *G.duodenalis* | (Garcia-Presedo et al., 2013) |
|  | 9 | 212 | *C.bovis*, *C.ryanae* | (Garcia-Presedo et al., 2013) | 2 | 48 | *G. duodenalis* ass. B | (Stojecki et al., 2015) |
|  | 2 | 22 |  | (Paziewska et al., 2007) | 1 | 22 |  | (Paziewska et al., 2007) |
|  |  |  |  |  | 1 | 649 | *G.duodenalis* | (Heitman et al., 2002) |
| Wild boar | 60 | 460 |  | (Nemejc et al., 2013) | 4 | 27 | *G.duodenalis* Ass. B | (Stojecki et al., 2015) |
|  | 33 | 193 |  | (Nemejc et al., 2012) | 4 | 381 |  | (Castro-Hermida et al., 2011) |
|  | 30 | 381 | *C. suvis, C.scrovarum* | (Castro-Hermida et al., 2011) |  |  |  |  |
| Aquatic birds | 18 | 308 | *C. parvum* | (Majewska et al., 2008) | 23 | 308 | *G. lamblia* | (Majewska et al., 2008) |
|  | 6 | 103 | *C. parvum* | (Plutzer and Tomor, 2009) | 6 | 103 | *G. duodenalis* | (Plutzer and Tomor, 2009) |

Table 2. Polynomial regression equations for the discharges and volumes of the backwater river, and the volumes and areas of inundation at the study site (Figure 2).

| Water quantity variable | Unit | n | Regression equation | R^2^ |
| --- | --- | --- | --- | --- |
| $Q_{bw}$ | M^3^/s | 29 | $3.163537\times{10}^{-6}Q_{r}^{2}+1.159649\times{10}^{-3}Q_{r}-18.70334$ | 0.99 |
| $V_{bw}$ | M^3^ | 48 | $2.01709904944854\times{10}^{-9}Q_{r}^{4}+ 2.07222626370030\times{10}^{-5}Q_{r}^{3}- 0.342891723249476\times Q_{r}^{2}+ 1236.45924269679\times Q_{r}- 901361.769680643$ | 0.99 |
| $A_{fl}$ | M^2^ | 48 | $1.48077257000492\times{10}^{-8}Q_{r}^{4}-1.83193932391713\times{10}^{-4}Q_{r}^{3}+ 0.834100479674070\times Q_{r}^{2}-1653.89413571683 \times Q_{r}+ 1852433.86553703$ | 0.99 |
| $V_{fl}$ | M^3^ | 48 | $6.00670232605541\times{10}^{-8}Q_{r}^{4}-8.55478579940220\times{10}^{-4}Q_{r}^{3}+ 4.46155449074763\times Q_{r}^{2}-1.00346048934779\times{10}^{4}\times Q_{r}+8.63000921932283\times{10}^{6}$ | 0.99 |

Table 3 Input parameter settings in QMRAcatch for calculating the surface runoff volume P_Q_ and changes in soil moisture as described by (Blöschl et al., 2008), eqs. 1-8: melt factor D, non-linearity parameter β, parameter limiting potential evaporation L_p_, lower (T_S_) and upper (T_R_) threshold temperature, melt temperature T_m_, and maximum soil moisture storage L_S_. Values were taken from Demeter et al. (2021).

| D | β | L_P_ | T_S_ | T_R_ | T_m_ | L_S_ |
| --- | --- | --- | --- | --- | --- | --- |
| mm/d/K | - | - | °C | °C | °C | mm |
| 2.0 | 2.1 | 0.5 | -0.8 | 0.8 | 0.3 | 180 |

Table 4. Reported first-order decay rates in water used in this study.

| **(Sero)type or qPCR assay** | **MST target or strain** | **Lighting condition** | **Water type** | **Temp (°C)** | **Enumeration method** | **k**  **(d^-1^)** | **Reference** |
| --- | --- | --- | --- | --- | --- | --- | --- |
| HF183 SYBR | Human | Natural light | Fresh, in situ | 15.8 | qPCR | -0.29 | (Ahmed et al., 2014) |
| HF183 target region (547 bp) | Human | Natural light | Fresh, in situ | 28 | qPCR | -3.03 | (He et al., 2016) |
| HF183 target region (547 bp) | Human | Natural light | Fresh, in situ | 12.7 | qPCR | -0.66 | (He et al., 2016) |
| HF183/BFDrev | Human | Natural light | Fresh, in situ | 26.5 | qPCR | -2.19 | (Balleste et al., 2018) |
| HF183/BFDrev | Human | Natural light | Fresh, in situ | 11.5 | qPCR | -0.71 | (Balleste et al., 2018) |
| HF183 SYBR | Human | Dark | Fresh, in lab | 25.2 | qPCR | -2.13 | (Dick et al., 2010) |
| HF183 SYBR | Human | Artificial sunlight | Fresh, in lab | 28.3 | qPCR | -2.69 | (Dick et al., 2010) |
| HF183 SYBR | Human | Artificial sunlight | Fresh, in lab | 15.0 | qPCR | -1.82 | (Dick et al., 2010) |
| HF183 target region (547 bp)/SYBR | Human | Natural light | Fresh | 11  (4-18) | qPCR | -0.74 | (Liang et al., 2012) |
| HF183 target region (547 bp)/SYBR | Human | Natural light | Fresh | 11  (4-18) | qPCR | -0.96 | (Liang et al., 2012) |
| HuBac | Human | Dark | Fresh | 18.5 | qPCR | -1.03 | (Jeanneau et al., 2012) |
| HF183 target region (547 bp) | Human | Natural sunlight | Fresh | 12.8 | qPCR | -1.20 | (Green et al., 2011) |
| HF183 target region (547 bp) | Human | Natural light | Fresh, in situ | 14.1 | qPCR | -1.36 | (Korajkic et al., 2014) |
| BacR | Ruminant | Natural light | Fresh, in situ | 5 | qPCR | -0.34 | (Sokolova et al., 2012) |
| BacR sewage isolate | Ruminant | Natural light | Fresh, in situ | 20 | qPCR | -1.64 | (Sokolova et al., 2012) |
| BacR | Ruminant | Natural light | Fresh, in situ | 6 | qPCR | -0.24 | (Sokolova et al., 2012) |
| BacR | Ruminant | Natural light | Fresh, in situ | 22.5 | qPCR | -3.29 | (Tambalo et al., 2012) |
| BacR | Ruminant | Natural light | Fresh, in situ | 22.5 | qPCR | -0.82 | (Tambalo et al., 2012) |
| BacCow | Ruminant | Natural light | Fresh | 14 | qPCR | -0.48 | (Bae and Wuertz, 2015) |
| Rum2Bac | Ruminant | Natural light | Fresh | 26.5 | qPCR | -1.55 | (Balleste et al., 2018) |
| Rum2Bac | Ruminant | Natural light | Fresh | 11.5 | qPCR | -0.50 | (Balleste et al., 2018) |
| BacR | Ruminant | Dark | Fresh | 5 | qPCR | -0.37 | (Sokolova et al., 2012) |
| BacR | Ruminant | Dark | Fresh | 20 | qPCR | -1.10 | (Sokolova et al., 2012) |
| BacR | Ruminant | Dark | Fresh | 6 | qPCR | -0.22 | (Sokolova et al., 2012) |
| Cow Bacteroidales | Ruminant | Dark | Fresh | 25 | qPCR | -0.88 | (Bae and Wuertz, 2012) |
| BacCow | Ruminant | Dark | Fresh | 14 | qPCR | -0.71 | (Bae and Wuertz, 2015) |
| CowBac2 | Ruminant | Dark | Fresh | 4 | Real-time PCR | -0.14 | (Okabe and Shimazu, 2007) |
| CowBac2 | Ruminant | Dark | Fresh | 10 | Real-time PCR | -0.74 | (Okabe and Shimazu, 2007) |
| CowBac2 | Ruminant | Dark | Fresh | 20 | Real-time PCR | -1.40 | (Okabe and Shimazu, 2007) |
| CowBac2 | Ruminant | Dark | Fresh | 30 | Real-time PCR | -1.59 | (Okabe and Shimazu, 2007) |
| Pig-2-Bac | Boar | Dark | Fresh | 18 | Real-time PCR | -1.21 | (Solecki et al., 2011) |
| Pig-2-Bac | Boar | Dark | River water | 20 | Real-time PCR | -1.21 | (Marti et al., 2011) |
| Pig-2-Bac | Boar | Dark | River water | 4 | Real-time PCR | -0.12 | (Marti et al., 2011) |
| Pig-2-Bac | Boar | Natural light | Fresh, in situ | 28 | qPCR | -2.56 | (He et al., 2016) |
| Pig-2-Bac | Boar | Natural light | Fresh, in situ | 12.7 | qPCR | -0.92 | (He et al., 2016) |
| Pig-2-Bac | Boar | Natural light | Fresh, in situ | 26.5 | qPCR | -1.44 | (Balleste et al., 2018) |
| Pig-2-Bac | Boar | Natural light | Fresh, in situ | 11.5 | qPCR | -0.79 | (Balleste et al., 2018) |
| Pig-2-Bac | Boar | Dark | River water | 4 | Real-time PCR | -0.12 | (Okabe and Shimazu, 2007) |
| Pig-2-Bac | Boar | Dark | River water | 10 | Real-time PCR | -0.62 | (Okabe and Shimazu, 2007) |
| Pig-2-Bac | Boar | Dark | River water | 20 | Real-time PCR | -0.97 | (Okabe and Shimazu, 2007) |
| Pig-2-Bac | Boar | Dark | River water | 30 | Real-time PCR | -1.13 | (Okabe and Shimazu, 2007) |
| Bac-Pre1 | Bird | Dark | River water | 4 | Real-time PCR | -0.14 | (Okabe and Shimazu, 2007) |
| Bac-Pre1 | Bird | Dark | River water | 10 | Real-time PCR | -0.55 | (Okabe and Shimazu, 2007) |
| Bac-Pre1 | Bird | Dark | River water | 20 | Real-time PCR | -1.36 | (Okabe and Shimazu, 2007) |
| Bac-Pre1 | Bird | Dark | River water | 30 | Real-time PCR | -1.63 | (Okabe and Shimazu, 2007) |
| *Giardia* | *G. muris* | Natural light | Fresh (lake/river) | 18 | Microscopy | -0.30 | (de Regnier et al., 1989) |
| *Giardia* | *G. muris* | Natural light | Fresh (lake/river) | 7 | Microscopy | -0.01 | (de Regnier et al., 1989) |
| *Giardia* | *G. muris* | Natural light | Fresh (lake/river) | 7 | Microscopy | -0.16 | (de Regnier et al., 1989) |
| *Giardia* | *G. muris* | Natural light | Fresh (lake/river) | 3.1 | Microscopy | -0.02 | (de Regnier et al., 1989) |
| *Giardia* | *G. muris* | Natural light | Fresh (lake/river) | 3.1 | Microscopy | -0.04 | (de Regnier et al., 1989) |
| *Giardia* | *G. muris* | Natural light | Fresh (lake/river) | 23 | Microscopy | -0.66 | (de Regnier et al., 1989) |
| *Giardia* | *G. muris* | Natural light | Fresh (lake/river) | 2 | Microscopy | -0.02 | (de Regnier et al., 1989) |
| *Giardia* | *G. muris* | Natural light | Fresh (lake/river) | 2 | Microscopy | -0.01 | (de Regnier et al., 1989) |
| *Giardia* | *G. muris* | Natural light | Fresh (lake/river) | 2 | Microscopy | -0.04 | (de Regnier et al., 1989) |
| *Cryptosporidium* | *C. parvum* | Dark | Fresh reservoir | 5 | Hemacytometer | -0.002 | (Ives et al., 2007) |
| *Cryptosporidium* | *C. parvum* | Dark | Fresh reservoir | 22 | Hemacytometer | -0.045 | (Ives et al., 2007) |
| *Cryptosporidium* | *C. parvum* | Dark | Fresh reservoir | 30 | Hemacytometer | -0.20 | (Ives et al., 2007) |
| *Cryptosporidium* | *C. parvum* | Dark | Fresh reservoir | 5 | Hemacytometer | -0.004 | (Ives et al., 2007) |
| *Cryptosporidium* | *C. parvum* | Dark | Fresh reservoir | 22 | Hemacytometer | -0.066 | (Ives et al., 2007) |
| *Cryptosporidium* | *C. parvum* | Dark | Fresh reservoir | 30 | Hemacytometer | -0.18 | (Ives et al., 2007) |

Ahmed, W., Gyawali, P., Sidhu, J.P.S., and Toze, S. (2014). Relative inactivation of faecal indicator bacteria and sewage markers in freshwater and seawater microcosms. *Letters in Applied Microbiology* 59(3)**,** 348-354. doi: 10.1111/lam.12285.

Bae, S., and Wuertz, S. (2012). Survival of Host-Associated <span class="named-content genus-species" id="named-content-1">Bacteroidales</span> Cells and Their Relationship with <span class="named-content genus-species" id="named-content-2">Enterococcus</span> spp., <span class="named-content genus-species" id="named-content-3">Campylobacter jejuni</span>, <span class="named-content genus-species" id="named-content-4">Salmonella enterica</span> Serovar Typhimurium, and Adenovirus in Freshwater Microcosms as Measured by Propidium Monoazide-Quantitative PCR. *Applied and Environmental Microbiology* 78(4)**,** 922-932. doi: 10.1128/aem.05157-11.

Bae, S., and Wuertz, S. (2015). Decay of host-associated Bacteroidales cells and DNA in continuous-flow freshwater and seawater microcosms of identical experimental design and temperature as measured by PMA-qPCR and qPCR. *Water Research* 70**,** 205-213. doi: <https://doi.org/10.1016/j.watres.2014.10.032>.

Balleste, E., Garcia-Aljaro, C., and Blanch, A.R. (2018). Assessment of the decay rates of microbial source tracking molecular markers and faecal indicator bacteria from different sources. *Journal of Applied Microbiology* 125(6)**,** 1938-1949. doi: 10.1111/jam.14058.

Blöschl, G., Reszler, C., and Komma, J. (2008). A spatially distributed flash flood forecasting model. *Environmental Modelling & Software* 23(4)**,** 464-478. doi: <https://doi.org/10.1016/j.envsoft.2007.06.010>.

Castro-Hermida, J.A., Garcia-Presedo, I., Gonzalez-Warleta, M., and Mezo, M. (2011). Prevalence of Cryptosporidium and Giardia in roe deer (Capreolus capreolus) and wild boars (Sus scrofa) in Galicia (NW, Spain). *Veterinary Parasitology* 179(1-3)**,** 216-219. doi: 10.1016/j.vetpar.2011.02.023.

de Regnier, D.P., Cole, L., Schupp, D.G., and Erlandsen, S.L. (1989). Viability of Giardia Cysts Suspended in Lake, River, and Tap Water. *Applied and Environmental Microbiology* 55(5)**,** 1223-1229.

Demeter, K., Derx, J., Komma, J., Parajka, J., Schijven, J., Sommer, R., et al. (2021). Modelling the interplay of future changes and wastewater management measures on the microbiological river water quality considering safe drinking water production. *Science of The Total Environment* 768**,** 144278. doi: <https://doi.org/10.1016/j.scitotenv.2020.144278>.

Dick, L.K., Stelzer, E.A., Bertke, E.E., Fong, D.L., and Stoeckel, D.M. (2010). Relative Decay of Bacteroidales Microbial Source Tracking Markers and Cultivated Escherichia coli in Freshwater Microcosms. *Applied and Environmental Microbiology* 76(10)**,** 3255-3262. doi: 10.1128/Aem.02636-09.

Dorner, S.M., Huck, P.M., and Slawson, R.M. (2004). Estimating potential environmental loadings of Cryptosporidium spp. and Campylobacter spp. from livestock in the Grand River Watershed, Ontario, Canada. *Environmental Science & Technology* 38(12)**,** 3370-3380. doi: 10.1021/es035208+.

Feng, Y.Y. (2010). Cryptosporidium in wild placental mammals. *Experimental Parasitology* 124(1)**,** 128-137. doi: 10.1016/j.exppara.2008.11.005.

Garcia-Presedo, I., Pedraza-Diaz, S., Gonzalez-Warleta, M., Mezo, M., Gomez-Bautista, M., Ortega-Mora, L.M., et al. (2013). The first report of Cryptosporidium bovis, C. ryanae and Giardia duodenalis sub-assemblage A-II in roe deer (Capreolus capreolus) in Spain. *Veterinary Parasitology* 197(3-4)**,** 658-664. doi: 10.1016/j.vetpar.2013.07.002.

Green, H.C., Shanks, O.C., Sivaganesan, M., Haugland, R.A., and Field, K.G. (2011). Differential decay of human faecal Bacteroides in marine and freshwater. *Environmental Microbiology* 13(12)**,** 3235-3249. doi: 10.1111/j.1462-2920.2011.02549.x.

He, X.W., Liu, P., Zheng, G.L., Chen, H.M., Shi, W., Cui, Y.B., et al. (2016). Evaluation of five microbial and four mitochondrial DNA markers for tracking human and pig fecal pollution in freshwater. *Scientific Reports* 6. doi: ARTN 35311

10.1038/srep35311.

Heitman, T.L., Frederick, L.M., Viste, J.R., Guselle, N.J., Morgan, U.M., Thompson, R.C.A., et al. (2002). Prevalence of *Giardia* and *Cryptosporidium* and characterization of *Cryptosporidium spp*. isolated from wildlife, human, and agricultural sources in the North Saskatchewan River Basin in Alberta, Canada. *Canadian Journal of Microbiology* 48(6)**,** 530-541. doi: 10.1139/W02-047.

Hinney, B., Gottwald, M., Moser, J., Reicher, B., Schafer, B.J., Schaper, R., et al. (2017). Examination of anonymous canine faecal samples provides data on endoparasite prevalence rates in dogs for comparative studies. *Veterinary Parasitology* 245**,** 106-115. doi: 10.1016/j.vetpar.2017.08.016.

Ives, R.L., Kamarainen, A.M., John, D.E., and Rose, J.B. (2007). Use of cell culture to assess Cryptosporidium parvum survival rates in natural groundwaters and surface waters. *Applied and Environmental Microbiology* 73(18)**,** 5968-5970. doi: 10.1128/Aem.00347-07.

Jeanneau, L., Solecki, O., Wery, N., Jarde, E., Gourmelon, M., Communal, P.Y., et al. (2012). Relative Decay of Fecal Indicator Bacteria and Human-Associated Markers: A Microcosm Study Simulating Wastewater Input into Seawater and Freshwater. *Environmental Science & Technology* 46(4)**,** 2375-2382. doi: 10.1021/es203019y.

Korajkic, A., McMinn, B.R., Shanks, O.C., Sivaganesan, M., Fout, G.S., and Ashbolt, N.J. (2014). Biotic Interactions and Sunlight Affect Persistence of Fecal Indicator Bacteria and Microbial Source Tracking Genetic Markers in the Upper Mississippi River. *Applied and Environmental Microbiology* 80(13)**,** 3952-3961. doi: 10.1128/Aem.00388-14.

Liang, Z.B., He, Z.L., Zhou, X.X., Powell, C.A., Yang, Y.E., Roberts, M.G., et al. (2012). High diversity and differential persistence of fecal Bacteroidales population spiked into freshwater microcosm. *Water Research* 46(1)**,** 247-257. doi: 10.1016/j.watres.2011.11.004.

Majewska, A.C., Graczyk, T.K., Slodkowicz-Kowalsk, A., Tamang, L., Jedrzejewski, S., Zduniak, P., et al. (2008). The role of free-ranging, captive, and domestic birds of Western Poland in environmental contamination with Cryptosporidium parvum oocysts and Giardia lamblia cysts. *Parasitology Research* 104(5)**,** 1093-1099. doi: 10.1007/s00436-008-1293-9.

Marti, R., Mieszkin, S., Solecki, O., Pourcher, A.M., Hervio-Heath, D., and Gourmelon, M. (2011). Effect of oxygen and temperature on the dynamic of the dominant bacterial populations of pig manure and on the persistence of pig-associated genetic markers, assessed in river water microcosms. *Journal of Applied Microbiology* 111(5)**,** 1159-1175. doi: 10.1111/j.1365-2672.2011.05131.x.

Nemejc, K., Sak, B., Kvetonova, D., Hanzal, V., Janiszewski, P., Forejtek, P., et al. (2013). Cryptosporidium suis and Cryptosporidium scrofarum in Eurasian wild boars (Sus scrofa) in Central Europe. *Veterinary Parasitology* 197(3-4)**,** 504-508. doi: 10.1016/j.vetpar.2013.07.003.

Nemejc, K., Sak, B., Kvetonova, D., Hanzal, V., Jenikova, M., and Kvac, M. (2012). The first report on Cryptosporidium suis and Cryptosporidium pig genotype II in Eurasian wild boars (Sus scrofa) (Czech Republic). *Veterinary Parasitology* 184(2-4)**,** 122-125. doi: 10.1016/j.vetpar.2011.08.029.

Nikolic, A., Dimitrijevic, S., Katic-Radivojevic, S., Klun, I., Bobic, B., and Djurkovic-Djakovic, O. (2008). High prevalence of intestinal zoonotic parasites in dogs from Belgrade, Serbia - Short communication. *Acta Veterinaria Hungarica* 56(3)**,** 335-340. doi: 10.1556/AVet.56.2008.3.7.

Okabe, S., and Shimazu, Y. (2007). Persistence of host-specific Bacteroides-Prevotella 16S rRNA genetic markers in environmental waters: effects of temperature and salinity. *Applied Microbiology and Biotechnology* 76(4)**,** 935-944. doi: 10.1007/s00253-007-1048-z.

Overgaauw, P.A.M., van Zutphen, L., Hoek, D., Yaya, F.O., Roelfsema, J., Pinelli, E., et al. (2009). Zoonotic parasites in fecal samples and fur from dogs and cats in The Netherlands. *Veterinary Parasitology* 163(1-2)**,** 115-122. doi: 10.1016/j.vetpar.2009.03.044.

Paziewska, A., Bednarska, M., Nieweglowski, H., Karbowiakl, G., and Bajer, A. (2007). Distribution of Cryptosporidium and Giardia spp. in selected species of protected and game mammals from North-Eastern Poland. *Annals of Agricultural and Environmental Medicine* 14(2)**,** 265-270.

Plutzer, J., and Tomor, B. (2009). The role of aquatic birds in the environmental dissemination of human pathogenic Giardia duodenalis cysts and Cryptosporidium oocysts in Hungary. *Parasitology International* 58(3)**,** 227-231. doi: 10.1016/j.parint.2009.05.004.

Sokolova, E., Astrom, J., Pettersson, T.J.R., Bergstedt, O., and Hermansson, M. (2012). Decay of Bacteroidales Genetic Markers in Relation to Traditional Fecal Indicators for Water Quality Modeling of Drinking Water Sources. *Environmental Science & Technology* 46(2)**,** 892-900. doi: 10.1021/es2024498.

Solecki, O., Jeanneau, L., Jarde, E., Gourmelon, M., Marin, C., and Pourcher, A.M. (2011). Persistence of microbial and chemical pig manure markers as compared to faecal indicator bacteria survival in freshwater and seawater microcosms. *Water Research* 45(15)**,** 4623-4633. doi: 10.1016/j.watres.2011.06.012.

Sterk, A., Schijven, J., Husman, A.M.D., and de Nijs, T. (2016). Effect of climate change on runoff of Campylobacter and Cryptosporidium from land to surface water. *Water Research* 95**,** 90-102. doi: 10.1016/j.watres.2016.03.005.

Stojecki, K., Sroka, J., Caccio, S.M., Cencek, T., Dutkiewicz, J., and Kusyk, P. (2015). Prevalence and molecular typing of Giardia duodenalis in wildlife from eastern Poland. *Folia Parasitologica* 62. doi: UNSP 042

10.14411/fp.2015.042.

Tambalo, D.D., Fremaux, B., Boa, T., and Yost, C.K. (2012). Persistence of host-associated Bacteroidales gene markers and their quantitative detection in an urban and agricultural mixed prairie watershed. *Water Res* 46(9)**,** 2891-2904. doi: 10.1016/j.watres.2012.02.048.

Trogu, T. (2015). *Giardia, Cryptosporidium and Eimeria infections in alpine wild ungulates: epidemiological investigation and management implications.* doctoral degree, Universitá degli studi di Milano.

Zygner, W., Jaros, D., Skowronska, M., Bogdanowicz-Kamirska, M., and Wedrychowicz, H. (2006). [Prevalence of Giardia intestinalis in domestic dogs in Warsaw]. *Wiad Parazytol* 52(4)**,** 311-315.
